# ZL-1211 Exhibits Robust Antitumor Activity by Enhancing ADCC and Activating NK Cell–mediated Inflammation in CLDN18.2-High and -Low Expressing Gastric Cancer Models

**DOI:** 10.1158/2767-9764.CRC-22-0216

**Published:** 2022-09-07

**Authors:** Hiroyasu Konno, Tracey Lin, Renyi Wu, Xinchuan Dai, Shou Li, Grace Wang, Min Chen, Wenying Li, Lina Wang, Bee-Chun Sun, Zhen Luo, Tom Huang, Yuping Chen, John Zhang, Qiuping Ye, David Bellovin, Bing Wan, Lishan Kang, Christopher Szeto, Karl Hsu, Omar Kabbarah

**Affiliations:** 1 Zai Lab (US) LLC, Menlo Park, California.; 2 Zai Lab (Shanghai) Co., Ltd. Pudong, Shanghai, P.R. China.

## Abstract

CLDN18.2 (Claudin18.2)-targeting therapeutic antibodies have shown promising clinical efficacy in approximately 30% of gastric cancers expressing high levels of CLDN18.2 and less pronounced activity in low expressing malignancies. Here, we report that ZL-1211 is a mAb targeting CLDN18.2 engineered to promote enhanced antibody-dependent cellular cytotoxicity (ADCC) with the goal of achieving more potent activity in a wider spectrum of high- and low-CLDN18.2 expressing tumors. ZL-1211 demonstrated more robust *in vitro* ADCC activity than clinical benchmark not only in CLDN18.2-high but also CLDN18.2-low expressing gastric tumor cell lines. Greater antitumor efficacy was also observed in mouse xenograft models. Natural killer (NK) cell played critical roles in ZL-1211 efficacy and NK-cell depletion abrogated ZL-1211–mediated ADCC activity *in vitro*. ZL-1211 efficacy *in vivo* was also dependent on the presence of an NK compartment. Strikingly, NK cells strongly induced an inflammatory response in response to ZL-1211 treatment, including increased IFNγ, TNFα, and IL6 production, and were recruited into tumor microenvironment in patient-derived gastric tumors expressing CLDN18.2 upon ZL-1211 treatment to lyse the tumor cells. Taken together, our data suggest that ZL-1211 more effectively targets CLDN18.2-high gastric cancers as well as -low expressing malignancies that may not be eligible for treatment with the leading clinical benchmark by inducing enhanced ADCC response and activating NK cells with robust inflammation to enhance antitumor efficacy. Clinical activity of ZL-1211 is currently under evaluation in a phase I clinical trial (NCT05065710).

**Significance:**

ZL-1211, anti-CLDN18.2 therapeutic antibody can target CLDN18.2-high as well as -low gastric cancers that may not be eligible for treatment with clinical benchmark. ZL-1211 treatment induces NK-cell activation with robust inflammation to further activate antitumor immunity in tumor microenvironment.

## Introduction

Gastric cancer is one of the most common and deadly cancer worldwide. Surgical resection offers the highest likelihood of being curative in early-stage gastric cancer, but in many cases gastric tumor is diagnosed at late stage due to nonspecific presenting symptoms. Thus, the prognosis remains very poor. Chemotherapy is a first-line treatment for unresectable or metastatic gastric cancer (1); however, such patients still eventually suffer from disease progression, indicating that better therapeutic approaches should be developed to improve outcomes.

Molecularly targeted therapies are one promising treatment modality being evaluated for patients with gastric cancer, which includes antibodies such as trastuzumab (anti-HER2 antibody) and pembrolizumab (anti-PD-L1 antibody; ref. 1). More recently, CLDN18.2, which is a tight junction protein in gastric mucosa, has been evaluated in clinical trials as a target molecule for gastric cancer (2). In normal stomach, CLDN18.2 is inaccessible to mAbs because CLDN18.2 is buried in a tight junction supermolecule complex (2). However, because the tight junction structure is disrupted in gastric cancer, CLDN18.2 epitopes are exposed on tumor cell surfaces and therefore accessible to therapeutic antibodies (2). Importantly, targeting CLDN18.2 is expected to have limited off-target effects as it is expressed at very low levels in normal tissues outside of the stomach (2). Therefore, CLDN18.2 is a promising target for gastric cancer treatment without major concern in toxicity.

Zolbetuximab (IMAB362), which is a first-in-class chimeric IgG1 mAb for CLDN18.2, has already shown promising clinical efficacy in gastric and esophageal adenocarcinoma as monotherapy and in combination with chemotherapy agents (3–5). However, the significant clinical benefit was observed only in approximately 30% of patients with high and medium CLDN18.2 expression (IHC3+/2+) in ≥70% of tumor cells (4). Furthermore, the clinical benefit rate of monotherapy was 23% among the CLDN18.2-high and -medium patients (4). In contrast, patients with CLDN18.2 expression-low tumors (IHC1+) were excluded from treatment with IMAB362 because of lack of the clinical response (4, 5). Therefore, development of a therapeutic antibody targeting CLDN18.2 with more potent activity in a wider spectrum of CLDN18.2-positive tumors, that is, CLDN18.2-high as well as -low expressing tumors, would address an unmet medical need in gastric tumor treatment.

Here, we report ZL-1211, a humanized monoclonal IgG1 antibody to target not only CLDN18.2-high but also -low gastric cancers with better efficacy than the clinical leading benchmark antibody, IMAB362. ZL-1211 binds CLDN18.2 with greater binding affinity than benchmark, targeting low expression levels of CLDN18.2 on gastric cancer. In addition, we introduced mutations on the Fc-domain of ZL-1211 to enhance ADCC affected by natural killer (NK) cells. ZL-1211 exhibited more potent *in vitro* as well as *in vivo* efficacy in gastric tumor models than benchmark. The mutated Fc-domain can induce activation of NK cells more potently, which mounts robust inflammatory responses including production of IFNγ, TNFα, and IL6 secreted from NK cells to further enhance immune reaction in tumor microenvironment. We also discovered that ZL-1211 treatment recruits NK cells into tumor microenvironment in gastric patient-derived xenografts (PDX) expressing various levels of CLDN18.2, ranging from low to high. Thus, based on our preclinical findings, ZL-1211 may show better clinical efficacy in patients with CLDN18.2-expressing gastric cancer than clinical leading benchmark.

## Materials and Methods

### Bioinformatics

To compare CLDN18.2 isoform expression levels across tissue types, isoform-specific expression data for >8.7K patients across 32 tissue types were obtained for The Cancer Genome Atlas (TCGA) cohort via the Broad GDAC Firehose server (source: https://gdac.broadinstitute.org/). Raw transcript per million (TPM) values for the CLDN18.2 isoform (i.e., uc003ero.1 transcript) were normalized by log_2_(TPM+1) conversion. To approximate clinically relevant categories of CLDN18.2 expression within RNA sequencing (RNA-seq) data, thresholds were defined by identifying quartiles within gastric cancer and then applied across tissue types. To investigate whether enhancing NK cell–mediated ADCC can potentially benefit patients with CLDN18.2-expressing gastric cancer, we performed differential survival analysis between high and low NK-cell activity within patients in the top three quartiles of CLDN18.2 expression. To define NK-cell activity subgroups, CIBERSORT (RRID:SCR_016955) inferred immune activity scores were obtained from TCGA sources (source: https://api.gdc.cancer.gov/data/b3df502e-3594-46ef-9f94-d041a20a0b9a; ref. 6) and patients were assigned high or low NK-cell activity by thresholding on the median gastric NK-cell activation score. Up-to-date progression-free interval and overall survival (OS) information was obtained from Liu and colleagues (7) and significance of differential survival between NK-cell activity subgroups was tested using the log-rank test. To demonstrate that low NK cell activity is not underrepresented within clinically relevant CLDN18.2 expression subgroups, enrichment analysis of the overlap between NK cell-low and CLDN18.2 subgroups was performed by Fisher exact test.

### Generation and Characterization of ZL-1211

ZL-1211 is a humanized IgG1 mAb, which is discovered through mouse immunization followed by hybridoma screening. ZL-1211 specifically binds to CLDN18.2 on the cell surface and is engineered to carry mutations in the Fc region that drives enhanced ADCC and unchanged complement-dependent cytotoxicity (CDC). Benchmark analog was generated in-house using CHO-K1Q cells (QuaCell) according to the sequence from WHO Drug Information, Vol. 31, No. 2, 2017, Page 358. Binding assay: CHO and NUGC4 cells were stably transduced with lentivirus carrying CLDN18 isoforms. The CLDN18.2- or CLDN18.1-overexpressing cells were incubated with ZL-1211 or isotype control at 4°C for 1 hour. The cells were then washed three times with FACS buffer (PBS with 1.5% FBS), followed by incubation with 1.67 μg/mL Alexa fluor 647-labeled anti-human IgG F(abʹ)_2_ specific antibody (Jackson ImmunoResearch Laboratories) at 4°C for 30 minutes. After washing three times, the cells were analyzed by BD FACS Celesta (BD biosciences). ADCC: NUGC4-hCLDN18.2 cells were labeled with carboxyfluorescein succinimidyl ester (CFSE) at 37°C for 10 minutes. The labeled cells were incubated with peripheral blood mononuclear cell (PBMC) from health donors at 37°C for 4–5 hours in presence of ZL-1211 or isotype control (E:T = 40:1) and subsequently stained with propidium iodide (PI) at 4°C for 15 minutes. After washing twice, cell death was analyzed by BD FACS Celesta. ADCC with CD16A-expressing Jurkat reporter cell: 45 μL of 2 × 10^4^ effector cells (Jurkat-NFAT-Luc2-CD16a-V158 or F158) and 45 μL of 2 × 10^4^ CHO-hCLDN18.2 cells were seeded into 96-well plate. A total of 10 μL of ZL-1211 or isotype control was added into the well. The mixture was incubated at 37°C for 6 hours followed by incubation with Bright-Glo (Promega) for 15 minutes. The luminescence was measured by Spectramax M3 (Molecular Devices). CDC: MIA-PaCa2-hCLDN18.2 was established using lentivirus. A total of 50 μL of 5 × 10^4^ MIA-PaCa2-hCLDN18.2 cells were seeded into ultra-low binding U-bottom 96-well plate (Corning). A total of 25 μL of serial diluted antibodies was added into the well and then incubated at room temperature for 20 minutes. Subsequently, 25 μL of 40% human serum (Gemini) was added into the wells and then the plate was incubated at 37°C for 30 minutes. The cells were further stained with PI at 4°C for 30 minutes. After washing twice, cell death was assessed by BD FACS Celesta. ADCP: Human macrophage was induced from human PBMC in the presence of 50 ng/mL M-CSF (R&D Systems). A total of 1 × 10^4^ of Celltrace Violet (Thermo Fisher Scientific)-labeled macrophages and 1 × 10^4^ of CFSE-labeled NUGC4-hCLDN18.2 cells were cocultured in the presence of ZL-1211 for 2 hours. After washing twice, the cells were analyzed by BD FACS Celesta to observe phagocytosis. Celltrace Violet and CFSE double-positive cells were defined as phagocytosis. Binding to Fcγ receptors and complement: binding kinetics of ZL-1211 to Fcγ receptors and complement C1q were measured using Biacore T200 (Cytiva) and Octet RED96 (Sartorius), respectively. The assay was performed at Shanghai OPM Biosciences.

### Cancer Cell Lines

SNU601 (00601, RRID:CVCL_0101) and SNU620 (00620, RRID:CVCL_5079) were obtained from KCLB in 2020 and maintained in RPMI with 10% FBS. KATOIII (HTB-103, RRID:CVCL_0371) and SNU5 (CRL-5973, RRID:CVCL_0078) were purchased from ATCC in 2020 and maintained in Iscove's modified Dulbecco's medium with 20% FBS. MIA-PaCa2 (CRL-1420, RRID:CVCL_0428) was also obtained from ATCC in 2020 and maintained in DMEM with 10% FBS and 2.5% horse serum. NUGC4 (JCRB0834, RRID:CVCL_3082) was obtained from JCRB in 2020 and maintained in RPMI with 10% FBS. PATU8988S (ACC204, RRID:CVCL_1846) was obtained from DSMZ in 2020 and maintained in DMEM with 10% FBS. All culture medias and serums were obtained from Gibco. SNU601, SNU620, KATOIII, and SNU5 were transduced with lentivirus carrying a luciferase gene with puromycin resistance gene (BPS Bioscience, 79692-P). After transduction, 5 μg/mL of puromycin (Gibco, A1113803) was added to cell culture media to eliminate cells that do not express the luciferase gene. The luciferase-expressing cells were used for ADCC and CDC. Cell line authentication: the morphology of the cell lines was confirmed by comparing with pictures from vendors after receiving and thawing the cell lines. CLDN18.2 expression was also confirmed by qPCR and flow cytometry as shown in figures, which further ensured whether we received correct cell lines. Cell passaging and culture time: a large number of cells for each cell line was cultured to generate many frozen cell vials after receiving cell lines (passage number: less than 10; culture time: less than 1 month). Before experiments, the frozen cells were freshly thawed and cultured to have enough number of the cells for the experiments (culture time: less than 2 weeks). Typically, 3–5 times passages were enough to conduct the experiments and then the cells were discarded. *Mycoplasma* testing: the test was conducted on July 6, 2022, for SNU601, SNU620, KATOIII, SNU5, NUGC4, and MIA-PaCa2, and all the cell lines were negative. PATU8988S was not tested.

### IHC

CLDN18.2 IHC was developed and performed with CellCarta (Belgium) using CLDN18.2-specific antibody (Abcam, EPR19202). For gastric PDX tumors, IHC was performed on consecutive sections at Acepix Biosciences using the following antibodies: CLDN18.2 (ab222512, EPR19202, 1:1,000, Abcam), human IgG (ab109489, EPR4421, 1:1,000, Abcam), and NKp46 (ab233558, EPR23097-35, 1:1,000, Abcam). Formalin-fixed and paraffin-embedded (FFPE) samples were sectioned at 4 μm and stained on BOND RX autostainer using BOND Polymer Refine Detection kit (Leica Microsystems) following manufacturer's instruction. After antigen retrieval using BOND Epitope Retrieval Solution 1 (human IgG) or 2 (CLDN18.2, NKp46) at 100°C for 15 minutes, slides were incubated with primary antibody for 1 hour, followed by Refine detection kit polymer for 10 minutes, DAB for 5 minutes, and hematoxylin for 5 minutes. The stained slides were scanned using 3DHisTech Digital scanner and the digitalized images were analyzed manually or using VisioPharm, the quantitative image analysis software. For NKp46 IHC image analysis using VisioPharm, tumor area of the whole section was selected as region of interest after removing necrotic and empty regions, and NKp46-positive and -negative cells were identified. NKp46-positive cell density, that is, number of positive cells per mm^2^ tumor area, was determined for all the tumor sections and compared between groups.

### qPCR, SNP Genotyping

RNA was extracted from MIA-PaCa2 clones or gastric tumor cell lines (SNU601, SNU620, KATOIII, SNU5) using PureLink RNA Mini kit (12183018A, Invitrogen). RNA concentration was determined by NanoDrop 8000 Spectrophotometer (Thermo Fisher Scientific, RRID:SCR_018600). Isolated RNA was used to synthesize cDNA using SuperScript VILO cDNA Synthesis Kit (11754050, Invitrogen). CLDN18.2 (Hs0098430_m1, Applied Biosystems) or CLDN18.1 (Hs00981422_m1, Applied Biosystems) TaqMan primer was used to detect the transcripts with TaqMan Fast Advanced Master Mix (4444554, Applied Biosystems) using QuantStudio5 (Applied Biosystems, RRID:SCR_020240). GAPDH TaqMan primer (Hs02786624_g1, Applied Biosystems) was used for normalization. cDNA from human lung and stomach was purchased from Takara Bio (636742, 636746). To determine CD16A polymorphism, genome DNA was extracted from PBMC using PureLink Genome DNA kit (K182001, Thermo Fisher Scientific). CD16A polymorphism in the extracted genome DNA was tested using TaqMan Genotyping assay mix for CD16A SNP (C__25815666_10, SNP ID: rs396991, Applied Biosystems) with TaqMan Genotyping master mix (4371355, Applied Biosystems). QuantStudio5 was used to determine polymorphisms.

### Flow Cytometry

MIA-PaCa2 clones expressing human CLDN18.2 or gastric tumor cell lines (SNU601, SNU620, KATOIII, SNU5) were incubated with ZL-1211 (100 μg/mL) in stain buffer (554656, BD Biosciences) for 1 hour on ice. After washing with stain buffer, the cells were further incubated with anti-human IgG-APC (FAB110A, R&D Systems) in stain buffer for 30 minutes on ice. To confirm NK cell depletion, PBMC was incubated with flow panel including anti-CD3-PE (UCHT1, BioLegend, RRID:AB_2562047), anti-CD4-APC (SK3, BioLegend, RRID:AB_2028488), anti-CD8-APC-Cy7 (SK1, BioLegend, RRID:AB_2044005), anti-CD14-BV421 (MPHIP9, BD Biosciences), anti-CD16-BV605 (B73.1, BD Biosciences, RRID:AB_2650663), anti-CD19-PE-Cy7 (SJ25C1, BioLegend, RRID:AB_2564203), anti-CD45-BV510 (2D1, BioLegend, RRID:AB_2687377), and anti-CD56-FITC (B159, BD Biosciences). Before incubation with the flow panel, Fc blocker (422302, BioLegend) was used to avoid nonspecific antibody binding. To detect intracellular perforin in NK cells, anti-perforin-BV711 (dG9, BioLegend, RRID:AB_2687189) was used after fixation and permeabilization with Fixation/Permeablization Kit (554714, BD Biosciences). Fixable Viability Dye (eBioscience) was used to exclude dead cells. Subsequent data acquired from LSRFortessa X-20 (BD Biosciences, RRID:SCR_019600) or Northern Lights (Cytek) was analyzed using FlowJo (BD Biosciences, RRID:SCR_008520).

### ADCC, CDC

Adherent cells (MIA-PaCa2, SNU601) were seeded into either 96 well or 384 well, and incubated with human IgG1 (BE0297, Bio X Cell), ZL-1211, or benchmark antibody at the indicated concentration for 1 hour. In case of suspension cells (SNU620, KATOIII, SNU5), antibody incubation was initiated immediately after seeding the cells. Because KATOIII grows as adherent as well as suspension cell, we considered KATOIII as suspension cell and incubated both adherent and suspension fractions with the antibodies. To perform ADCC, human PBMC (70025, STEMCELL technology) or purified NK cells (70036, STEMCELL Technology) was used as effector cells and added to the well at the indicated E:T ratio after 1 hour incubation with antibody. After 24 hours (MIA-PaCa2) or 72 hours (gastric tumor cell lines), cell viability in MIA-PaCa2 was measured using CellTiter-Glo (G9242, Promega) or luciferase activity in gastric tumor cell lines was measured using ONE-Glo Luciferase Assay System (E6120, Promega). Luminescence was detected by EnVision (PerkinElmer) or CLARIOstar plus (BMG LABTECH). In case of CDC, human AB serum (BP2525100, Thermo Fisher Scientific) was used as a source of complement, but other procedures were almost same as described for ADCC, but incubation time after adding serum was 24 hours for both MIA-PaCa2 and gastric tumor cell lines. Compared with luminescence from cell without antibody, cell lysis levels were calculated.

### NK cell Depletion, Cytokine Detection

NK cells in PBMC were depleted using EasySep Mouse NK Cell Isolation Kit (19855, STEMCELL Technology). NK depletion was confirmed by flow cytometry. To measure inflammatory cytokines, supernatants were collected after incubation for ADCC assay. Cytokines in the supernatants were measured by V-PLEX Proinflammatory Panel 1 Human Kit [K15049D, Meso Scale Discovery (MSD)] using MESO QuickPlex SQ 120 (MSD, RRID:SCR_020304). The data were analyzed using MSD Discovery Workbench (MSD, RRID:SCR_019192).

### *In Vivo* Mouse Models

A total of 5 × 10^6^ cells of SNU620, KATOIII, or SNU5 were subcutaneously inoculated into Balb/c nude mouse (7–9 weeks old female, Charles River) at the right hind flank with 0.1 mL of 1:1 mixture of PBS and Matrigel (354263, Corning). After randomization, human IgG1 (BE0297, Bio X Cell), ZL-1211, or benchmark antibody at the indicated concentration was injected by intraperitoneal route once weekly and tumor volume was measured twice a week. SNU601 tumor models with Balb/c nude, NOD.SCID, or NCG mouse, and gastric PDX models with Balb/c nude mouse were performed at Crown Bioscience. Briefly, each mouse was inoculated subcutaneously in the right upper flank region with 2.5 × 10^7^ SNU601 cells in 0.1 mL of PBS mixed with Matrigel (1:1). 5 mg/kg of human IgG1 (C0001-5, Crown Bioscience) or ZL-1211 was injected by intraperitoneal route once weekly and tumor volume was measured three times a week. At the end of study, tumors were collected, and the single-cell suspension was accessed by flow cytometry to observe mouse immune cell infiltration into tumor microenvironment with a flow panel including anti-CD45-BV785 (30-F11, BioLegend, RRID:AB_2564590), anti-CD335-APC-FIRE750 (29A1.4, BioLegend, RRID:AB_2617041), anti-CD69-APC (H1.2F3, BioLegend, RRID:AB_492844), anti-Granzyme B-PE (QA16A02, BioLegend, RRID:AB_2687031), anti-CD11b-BB700 (M1/70, BD Biosciences), anti-F4/80-PE-Cy7 (BM8, BioLegend, RRID:AB_893478), anti-CD206-FITC (C068C2, BioLegend, RRID:AB_10900988), anti-IA-IE-Alexa Fluor 700 (M5/114.15.2, Biolegend, RRID:AB_493726), anti-Ly6C-BY421 (HK1.4, BioLegend), anti-LY6G-BV605 (1A8, BioLegend, RRID:AB_2565880), and cell viability dye (eFluor506, 65-0866-14, Invitrogen). Gastric PDX fragments from stock mice were harvested and used for inoculation into Balb/c nude mouse. Each mouse was inoculated subcutaneously in the right rear flank with the indicated PDX fragment (2–3 mm in diameter). A total of 50 mg/kg of human IgG1 (C0001-5, Crown Bioscience) or ZL-1211 was injected by intraperitoneal route three times a week and tumor volume was measured twice a week. Tumors were harvested at the end of study and subsequently tumor FFPE was generated for IHC test. Pharmacokinetic parameters in SNU620 tumor model were measured as following: Following repeated intraperitoneal administration of ZL-1211 at the doses of 0.1, 1, and 10 mg/kg/once weekly to female SNU620 tumor-bearing Balb/c nude mice, the serum samples were collected at the timepoints of 4, 24, 48, 72, and 168 hours (*n* = 2) after last dose and stored at −80°C until analysis. The analytic standards spiked with ZL-1211 and the serum samples were prepared using a 96-well ELISA reagent kit (MEDNA Scientific), and analyzed for the concentration of ZL-1211 using an Epoch Microplate Spectrophotometer (BioTek Instrument) at 450 nm. The serum concentration–time profiles of ZL-1211 were used to estimate the pharmacokinetic parameters with Phoenix WinNonlin software (version 8.2, Certara). The protocol and any amendment(s) or procedures involving the care and use of animals in this study were reviewed and approved by the Institutional Animal Care and Use Committee (IACUC) of CrownBio prior to execution. During the study, the care and use of animals were conducted in accordance with the regulations of the Association for Assessment and Accreditation of Laboratory Animal Care. All mouse modeling studies conducted in this study at Zai Lab were performed according to an approved IACUC protocol.

### Statistical Analysis

The *t* test or ANOVA test was performed to determine the statistical significance of differences between two or more variables. All statistical analyses and calculation of EC_50_ were carried out using GraphPad Prism 9 (RRID:SCR_002798).

### Data Availability Statement

Data are available upon reasonable request made to the corresponding author.

## Results

### ZL-1211 is a Novel Humanized Monoclonal IgG1 Antibody that Targets CLDN18.2-expressing Tumors

**FIGURE 1:**
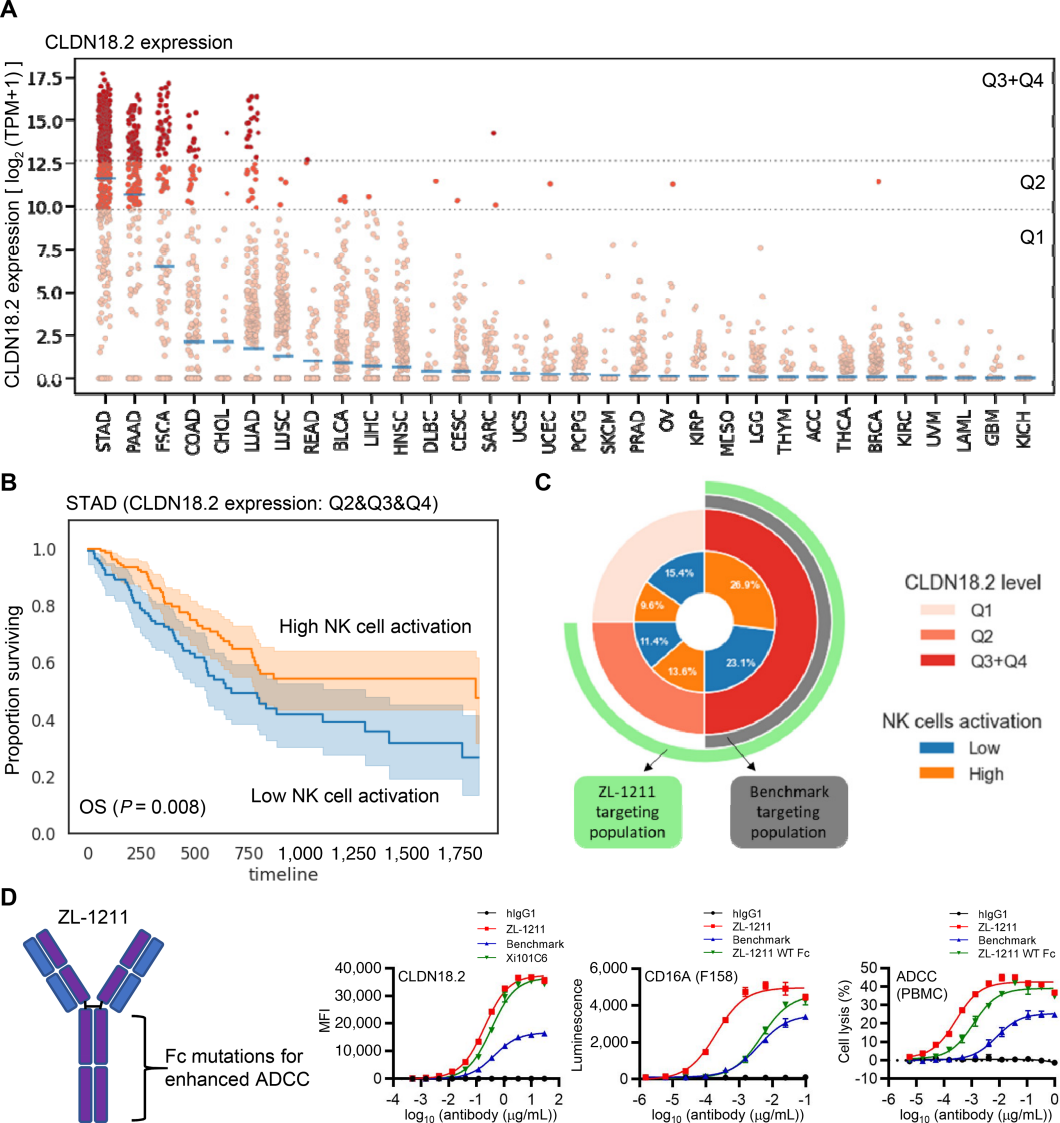
CLDN18.2 is an ideal gastric cancer cell surface marker and the mAb ZL-1211 was designed to target not only CLDN18.2-high but also -low tumors. **A,** CLDN18.2 expression levels for over 8.7K patients across 32 tissue types in TCGA. Individual patients are colored based on which clinically relevant subgroup they fall into: Above the 50th percentile in gastric cancer (red, Q3+4), above the 25th percentile, (orange, Q2), or below the 25th percentile (pink, Q1). Average expression levels per tissue type are shown as blue lines. **B,** Kaplan–Meier analysis of OS in CLDN18.2+ STAD patients subgrouped by level of inferred active NK cells, up to 5 years follow-up. Log-rank *P* value is shown in bottom left. **C,** Overlap of clinically relevant CLDN18.2 subgroups and low NK activity subgroups. **D,** Structure of ZL-1211 (left). ZL-1211 binding to CLDN18.2 (left graph) and CD16A (middle graph), and ADCC induced by ZL-1211 (right graph). Xi101C6 discovered during antibody screening can bind both CLDN18.1 and CLDN18.2. ZL-1211 WT Fc is an antibody without the Fc-mutations.

Human *CLDN18* has two isoforms, *CLDN18.1* and *CLDN18.2* (Supplementary Fig. S1A). Both isoforms consist of 261 amino acids, but the N-terminus (1–69aa) is different due to the first exon, which includes the first extracellular loop that is a target sequence for therapeutic antibodies. While *CLDN18.1* and *CLDN18.2* expression is limited to lung and stomach respectively among normal tissues, Sahin and colleagues showed that the *CLDN18.2* isoform specifically has increased expression in gastric, pancreatic, esophageal, lung, and colorectal tumors (8). By using TCGA dataset, we further confirmed that few tissue types across 32 TCGA indications ubiquitously express *CLDN18.2* isoform (Fig. 1A). Gastric stomach adenocarcinomas (STAD) have the most elevated level of *CLDN18.2* isoform expression (mean TPM: 3280.8), followed by pancreatic adenocarcinomas (mean TPM: 1730.0), esophageal carcinomas (ESCA; mean TPM: 95.0), and colon adenocarcinomas (mean TPM: 3.5). In ESCA, adenocarcinoma (mean TPM: 2569.9) expresses much higher *CLDN18.2* than squamous carcinoma (mean TPM: 2.6).

Reports have shown that IMAB362 induces ADCC to inhibit tumor cell growth (3, 8). NK cell is known to induce ADCC to lyse tumor cells upon therapeutic antibody binding (9, 10). Within gastric cancers, the level of CLDN18.2 expression was not associated with poor prognosis [above the 50th percentile in gastric cancer (red, Q3+4), above the 25th percentile, (orange, Q2), or below the 25th percentile (pink, Q1)] (Fig. 1A; Supplementary Fig. S1B). However, within CLDN18.2-positive gastric cancers (Q2+3+4), low NK activity was found to be a significant marker of poor prognosis (*P* = 0.008; Fig. 1B), suggesting NK cell recruitment and activation through therapeutic treatment may benefit this high-risk population. Furthermore, this low NK-activation population was equally represented between Q2 and Q3+4 CLDN 18.2 subgroups (OR = 0.9, *P* = 0.7; Fig. 1C), implying the opportunity for therapeutic treatment to activate and recruit NK cell is equivalent across the CLDN18.2-positive population.

Because CLDN18.2-low gastric tumor patients are not eligible for IMAB362 treatment (4, 5), we decided to generate a therapeutic antibody to target CLDN18.2-low gastric cancer (Q2) as well as CLDN18.2-medium/high cancer (Q3+4; Fig. 1C). In addition, to further improve treatment outcomes, we also considered activation of NK cell in gastric tumor microenvironment by the antibody treatment because low NK cell activation in gastric tumor correlates with poor prognosis (Fig. 1B). To achieve better efficacy and NK activation simultaneously, we generated a humanized monoclonal IgG1 antibody, ZL-1211 (Fig. 1D). During antibody screening, we selected clones with high affinity for CLDN18.2. In addition, we introduced mutations on ZL-1211 Fc-domain to enhance ADCC because the mutations are known to increase affinity to Fcγ receptors on immune effector cells such as NK cell (11–13). We hypothesized that ZL-1211 may activate NK cells upon binding to the Fcγ receptor, which potentially improves prognosis of patients with gastric cancer (Fig. 1B).

To compare ZL-1211 with the leading clinical benchmark (IMAB362), we also generated a benchmark analog antibody using a disclosed amino acid sequence as described in Materials and Methods. We could not directly compare the benchmark analog with clinical grade IMAB362. Instead, we decided to evaluate potency of the analog antibody by using EC_50_ of IMAB362 published on patent or scientific journals. EC_50_ of IMAB362 by ADCC for pancreatic tumor cell line PATU8988S is 50 to 500 ng/mL with PBMC from healthy donors (U.S. patent: US9770487B2). We evaluated ADCC capability by the benchmark analog for PATU8988S with PBMC (Supplementary Fig. S1C). The EC_50_ of the analog antibody was 379 ng/mL that was in the EC_50_ range for IMAB362. However, IMAB362 used in the patent might not be clinical grade antibody currently used in clinical trials. Therefore, we further looked for available data from clinical trials with IMAB362. In the phase I clinical trial, ADCC with PBMC from healthy donors or patients was evaluated using NUGC4 gastric tumor cell line and the median EC_50_ was approximately 200 ng/mL (3). We confirmed the benchmark analog induced ADCC for NUGC4 with EC_50_ = 84 ng/mL (Supplementary Fig. S1C). These results verified the benchmark analog generated in house is functionally comparable with IMAB362.

ZL-1211 specifically bound the CLDN18.2 isoform with better affinity than benchmark analog (Fig. 1D; Supplementary Fig. S1D). We confirmed the Fc-mutations did not affect interaction between ZL-1211 and CLDN18.2 because ZL-1211 wild-type (WT) Fc, which does not carry the mutations on the Fc-domain, bound CLDN18.2 similarly to ZL-1211 (Supplementary Fig. S1E). In addition to increased target affinity, the Fc-mutations dramatically enhanced binding affinity between ZL-1211 Fc-domain and Fcγ receptor type IIIA (CD16A) expressed on NK cells, and induced activation of downstream signaling more potently, compared with ZL-1211 WT Fc and benchmark (Fig. 1D; Supplementary Fig. S1F and S1H). CD16A has polymorphisms distributed within the normal population at amino acid reside 158 that is known to affect affinity with Fc-domain of antibody (14). Therefore, we also confirmed if ZL-1211 can interact with both high (158V/V) and low (158F/F) affinity CD16As. Both high (V158) and low (F158) affinity receptors bound ZL-1211 more strongly than ZL-1211 WT Fc or benchmark (Fig. 1D; Supplementary Fig. S1F and S1H).

One mechanism of action when using mAb for tumor treatment is ADCC, CDC, or antibody-dependent cellular phagocytosis (ADCP; ref. 15). To evaluate ADCC capability of ZL-1211, CLDN18.2-expressing NUGC4 cells were incubated with PBMC including NK cells in presence of ZL-1211, ZL-1211 WT Fc, or benchmark (Fig. 1D; Supplementary Fig. S1G). The results demonstrate that ZL-1211 induces robust ADCC-mediated tumor cell death compared with other two antibodies. Not only ADCC but also CDC and ADCP were induced by ZL-1211 more potently than benchmark (Supplementary Fig. S1G). There results indicate that ZL-1211 can be a more potent therapeutic antibody to target CLDN18.2-expressing tumors than leading clinical benchmark.

### ZL-1211 can Target not only CLDN18.2-high but also -low Expressing Tumor Cells

**FIGURE 2:**
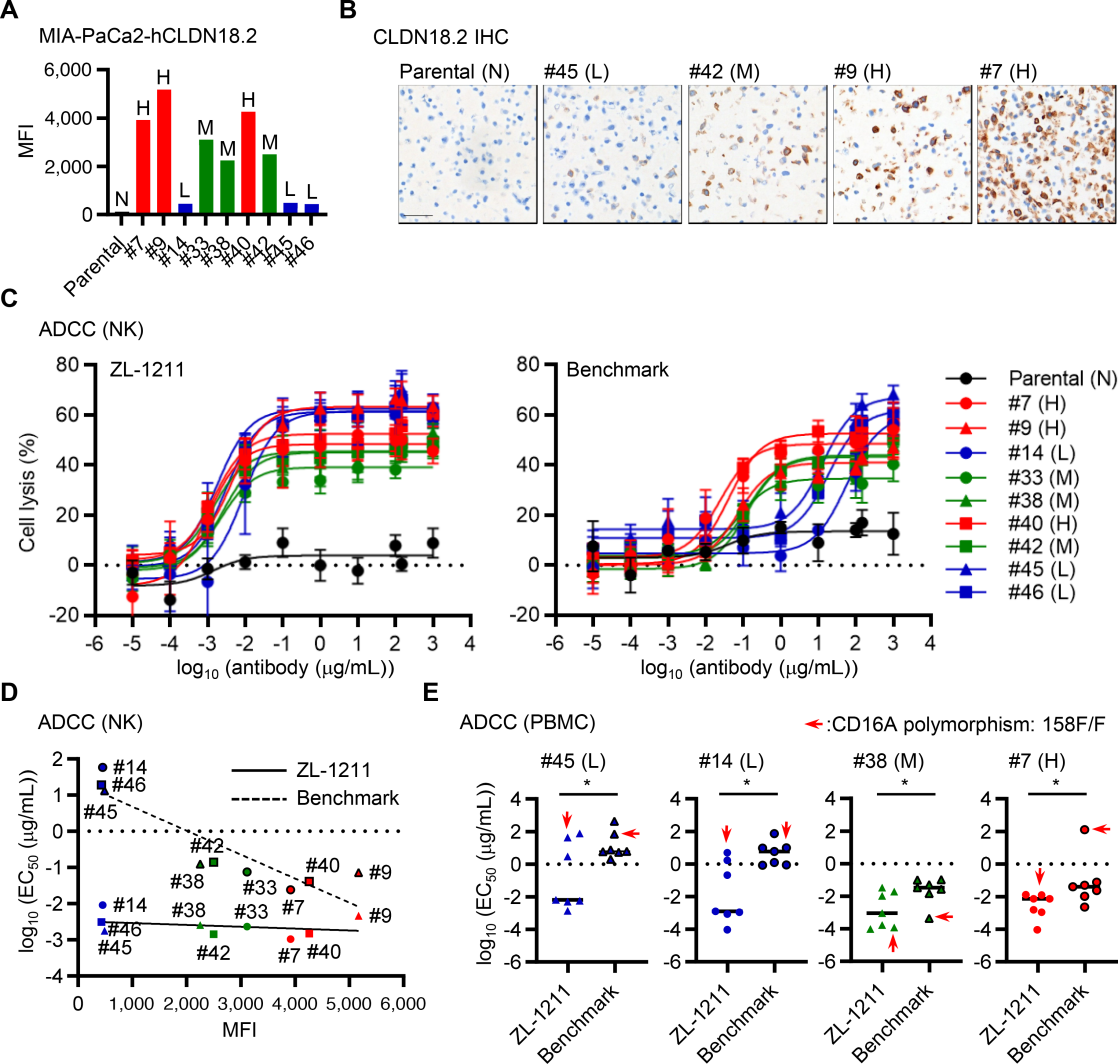
ZL-1211 exhibits robust ADCC not only in CLDN18.2-high but also -low tumor cells than benchmark. **A,** CLDN18.2 expression in MIA-PaCa2 clones. MFI indicates mean fluorescent intensity. H, M, L, or N indicates high, medium, low, or negative, respectively. **B,** CLDN18.2 expression in the isolated clones was further confirmed by CLDN18.2 IHC. Scale bar, 100 μm. **C,** ADCC with purified NK cells in presence of ZL-1211 or benchmark antibody. Effector to tumor cell ratio (E:T) was 5:1. **D,** Comparison of EC_50_ for NK-mediated ADCC with ZL-1211 or benchmark. **E,** ADCC with human PBMCs from seven healthy donors (E:T = 20:1). The EC_50_ for the indicated clones was compared. Among seven donors, two donors have 158V/V, four donors have 158F/V, and one donor has 158F/F. Arrowhead indicates a donor with 158F/F. *, *P* < 0.05.

We next tested whether ZL-1211 can target CLDN18.2-low tumors. We overexpressed human CLDN18.2 (hCLDN18.2) in the MIA-PaCa2 pancreatic tumor cell line, which is ordinarily CLDN18.2 negative and was used to evaluate IMAB362 efficacy *in vitro* and *in vivo* (16). The CLDN18.2-positive clones were isolated by cell sorting and the CLDN18.2 expression in each clone was confirmed by flow cytometer, qPCR, and CLDN18.2 IHC (Fig. 2A and B; Supplementary Fig. S2A and S2B). On the basis of the expression levels, we defined CLDN18.2-high (H), -medium (M), and -low (L) clones [parental: negative (N)]. The MIA-PaCa2-hCLDN18.2 clones and purified human NK cell (CD16A polymorphism: 158F/F) were cocultured with ZL-1211 or benchmark to measure ADCC activity (Fig. 2C). Although ZL-1211 could induce ADCC almost equally against all CLDN18.2-high, -medium, and -low clones, benchmark required higher antibody concentration to induce ADCC especially against CLDN18.2-low clones (Fig. 2C). Comparison of EC_50_ showed that ZL-1211 is more potent for all CLDN18.2-high, -medium, and -low clones than benchmark. Especially within CLDN18.2-low clones ZL-1211 was approximately 1,000-fold as potent as benchmark (Fig. 2D). We further evaluated ADCC capability of ZL-1211 with human PBMCs from seven healthy donors [CD16A polymorphism: 158F/F (1 donor), F/V (4 donors), V/V (2 donors)] (Fig. 2E). ZL-1211 induced tumor cell death more efficiently for all CLDN18.2-high, -medium, and -low clones than benchmark. We also attempted to characterize how CD16A polymorphism impacts ADCC activity with ZL-1211 treatment but did not observe an obvious trend regarding the presence of polymorphisms (Fig. 2E). With purified human NK cells from healthy donors harboring different CD16A polymorphisms (158F/F, F/V, V/V), we evaluated ADCC capability of ZL-1211, but all the polymorphisms showed almost equivalent EC_50_ with ZL-1211 (Supplementary Fig. S2C), suggesting that CD16A polymorphisms do not appear to impact on ADCC with ZL-1211 at least *in vitro*. We also evaluated ZL-1211 CDC capability with human serum (Supplementary Fig. S2D). While ZL-1211 could induce CDC-mediated tumor cell death in CLDN18.2-high and -medium clones at 0.1 μg/mL of antibody, benchmark needed much higher antibody concentration to induce CDC. These results clearly indicate that ZL-1211 can target not only CLDN18.2-high/medium but also -low expressing tumors by ADCC as well as CDC much more potently than benchmark.

### ZL-1211 Exhibits Robust Antitumor Activity by ADCC not only in CLDN18.2-high but also -low Expressing Gastric Cancer Models

**FIGURE 3:**
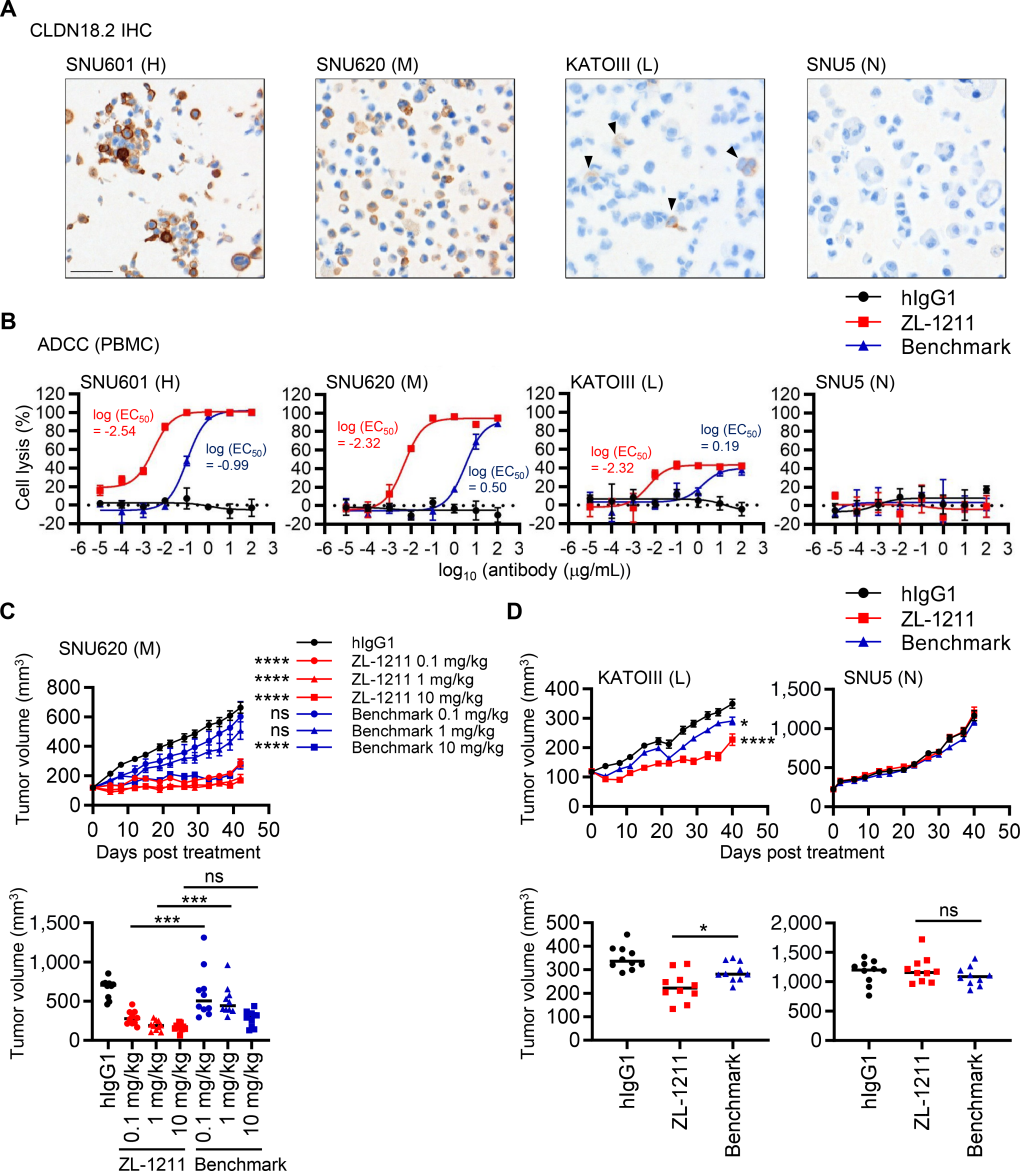
ZL-1211 inhibits CLDN18.2-high, -medium, and -low gastric tumor cell growth by inducing robust ADCC. **A,** CLDN18.2 IHC for gastric tumor cell lines. H, M, L, or N indicates high, medium, low, or negative, respectively. Scale bar, 100 μm. **B,** ADCC with PBMC from health donors (*n* = 3) at E:T = 20:1. The result with one donor is shown here as representative of three donors. All the three donors showed similar ADCC activity. **C,** ZL-1211 suppressed SNU620 growth *in vivo*. SNU620 cells were subcutaneously inoculated into Balb/c nude mice (*n* = 10). 10 mg/kg of control antibody (hIgG1) or the indicated concentration of ZL-1211 or benchmark antibody was injected by intraperitoneal route once weekly. At day 42, the tumor volume was compared (bottom graph). **D,** ZL-1211 suppressed KATOIII but not SNU5 growth *in vivo*. KATOIII or SNU5 was subcutaneously inoculated into Balb/c nude mice (*n* = 10). 10 mg/kg of the indicated antibodies was injected by intraperitoneal route once weekly. At day 40, the tumor volume was compared (bottom graph). ****, ***, *, or ns indicates *P* < 0.0001, *P* < 0.001, *P* < 0.05, or not significant, respectively.

We next evaluated the potency of ZL-1211 in gastric tumor using *in vitro* and *in vivo* models because CLDN18.2 is highly expressed in gastric tumors (Fig. 1A). We utilized a panel of gastric tumor cell lines that endogenously express CLDN18.2, which more accurately reflect target levels in human gastric tumors than engineered overexpressing models. To identify cell line models that express CLDN18.2, we obtained RNA-seq expression values from the DepMap database (https://depmap.org/portal/) for gastric tumor cell lines (Supplementary Fig. S3A). On the basis of the expression levels of *CLDN18*, we chose SNU601, SNU620, KATOIII, and SNU5. Because the downloaded RNA-seq data do not differentiate CLDN18 isoforms, we confirmed by qPCR that SNU601, SNU620, and KATOIII express only *CLDN18.2* but not *CLDN18.1*, and SNU5 does not express both isoforms (Supplementary Fig. S3B). CLDN18.2 expression was further confirmed by CLDN18.2 IHC and flow cytometry (Fig. 3A, Supplementary Fig. S3B–S3D) and we defined SNU601, SNU620, KATOIII, and SNU5 as CLDN18.2-high (H), -medium (M), -low (L), and -negative (N) gastric tumor cell lines. Except for SNU5, ZL-1211 could induce more robust ADCC with PBMC for SNU601, SNU620, and KATOIII than benchmark (Fig. 3B). It should be noted that KATOIII expresses minimal amount of CLDN18.2, which was hardly detected by qPCR, flow cytometry, and CLDN18.2 IHC (Fig. 3A; Supplementary Fig. S3B–S3D). We also confirmed that ZL-1211 induces more potent CDC in SNU601 and SNU620 than benchmark (Supplementary Fig. S3E). These results indicate that ZL-1211 is more potent antibody for gastric tumors endogenously expressing not only high but also low levels of CLDN18.2 than benchmark.

We also evaluated *in vivo* efficacy of ZL-1211 in comparison to benchmark (Fig. 3C and D). SNU620 cells were inoculated into Balb/c nude mice and treated by ZL-1211 or benchmark once a week. ZL-1211 could suppress tumor growth even at 0.1 and 1 mg/kg of antibody, but benchmark required 10 mg/kg to achieve inhibition of tumor growth although exposure of benchmark was about twice as high as ZL-1211 (Supplementary Fig. S3F). KATOIII tumor growth was also inhibited by ZL-1211 more potently than benchmark while both antibodies failed to inhibit CLDN18.2-negative SNU5 growth (Fig. 3D). These results indicate that ZL-1211 can target not only CLDN18.2-high but also -low gastric tumors and exhibit better *in vivo* efficacy than benchmark.

### ZL-1211 Induces Robust NK Cell–mediated ADCC

**FIGURE 4:**
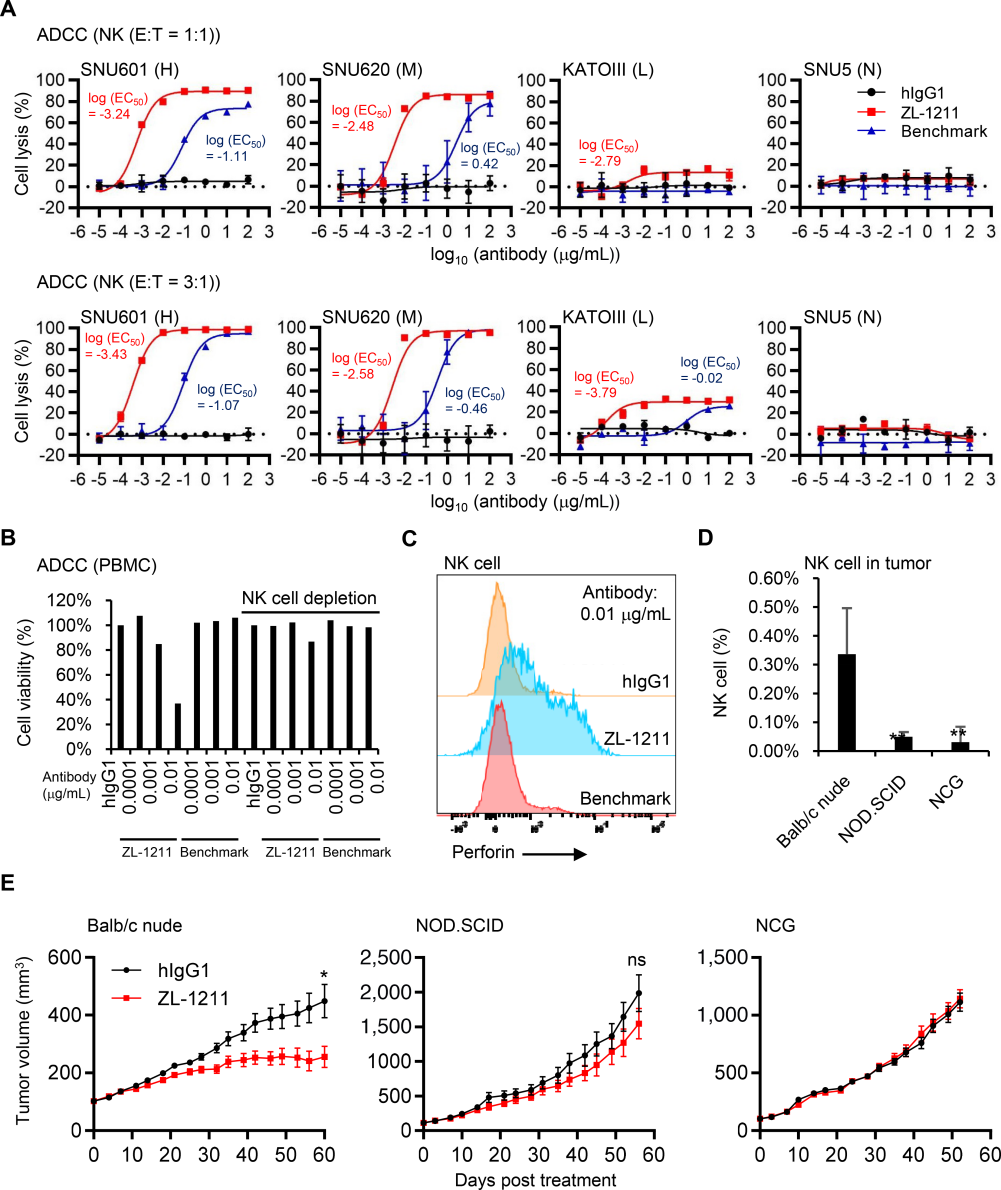
ZL-1211 induces robust ADCC in a NK cell–dependent manner. **A,** ADCC with purified NK cells at E:T = 1:1 or 3:1 in presence of ZL-1211 or benchmark. **B,** NK cells were depleted from human PBMC using anti-CD56 magnetic beads. The NK-depleted PBMC lost ADCC capability for SNU601. **C,** NK cell was activated by ZL-1211. SNU601 was incubated with PBMC in presence of ZL-1211 or benchmark for 3 days. Suspension cells were collected and then stained with antibodies for flow cytometry. Intracellular perforin levels were measured in NK-cell fraction. **D,***In vivo* NK cell level in SNU601 tumor. Tumor-infiltrating NK cell levels in CD45^+^ were determined by flow cytometry. **E,** ZL-1211 could show efficacy only in NK-intact mouse model. SNU601 cells were subcutaneously inoculated into Balb/c nude, NOD.SCID, or NCG mice (*n* = 10). 5 mg/kg of control antibody (hIgG1) or ZL-1211 was injected by intraperitoneal route once weekly. * or ns indicates *P* < 0.05 or not significant, respectively.

NK cell is known as an effector cell to induce ADCC with therapeutic antibodies (11–13). In addition, within CLDN18.2-positive gastric cancers (Q2+3+4), low NK activity was found to be significant marker of poor prognosis (Fig. 1B), suggesting activation of NK cell may be beneficial for patients with gastric tumor. Thus, we decided to evaluate whether NK cell is required for ZL-1211 efficacy. Purified human NK cells induced more robust ADCC for gastric tumor cell line SNU601, SNU620, and KATOIII with ZL-1211 than benchmark (Fig. 4A). It should be noted that ZL-1211 is capable to mount ADCC activity for KATOIII expressing minimal amount of CLDN18.2 even with low effector:tumor (E:T) ratio (1:1) while benchmark did not show efficacy at the E:T ratio, suggesting that ZL-1211 may be able to be used for treatment of CLDN18.2-low as well as NK-low gastric tumors. We will further evaluate the ZL-1211 potency for CLDN18.2-low and/or NK-low tumors in our clinical trial.

We further confirmed that NK cell is required to induce ADCC with ZL-1211 because ZL-1211 failed to activate ADCC with NK-depleted PBMC (Fig. 4B; Supplementary Fig. S4A). In addition, we discovered NK-cell activation by ZL-1211 because perforin, which is released from activated NK cell to destroy cancer cells, dramatically increased (Fig. 4C). These results clearly indicate that NK cell is required for ZL-1211 efficacy and ZL-1211 can activate NK cell to lyse gastric tumor cells.

Requirement of NK cell for *in vivo* ZL-1211 efficacy was also evaluated (Fig. 4D and E). We decided to test three mouse models (Balb/c nude, NOD.SCID, NCG) with ZL-1211 because Balb/c nude mice are known to have intact NK cells while NK cell partially loses effector function in NOD.SCID and NCG lacks NK cell (17, 18). SNU601 cells were inoculated into the indicated mouse models and treated by 5 mg/kg of ZL-1211 once a week. To accurately interpret results, we confirmed NK cell levels in tumor and spleen of the tested mouse models (Fig. 4D; Supplementary Fig. S4B). NK cells were detected by flow cytometry in tumor as well as spleen from Balb/c nude mice. In contrast, NK-cell fraction was easily detected in spleen but not in tumor from NOD.SCID mice. In NCG mice, both spleen and tumor did not contain NK-cell fraction. Consistent with the NK cell levels in tumors, ZL-1211 suppressed SNU601 tumor growth in Balb/c nude mice while NOD.SCID mice exhibited minimal response, and NCG mice did not display any evidence of *in vivo* efficacy (Fig. 4E). These results clearly indicate that NK cell is required for ZL-1211 *in vivo* efficacy to suppress gastric tumor growth.

### ZL-1211 Induces Robust NK Cell–mediated Inflammation

**FIGURE 5:**
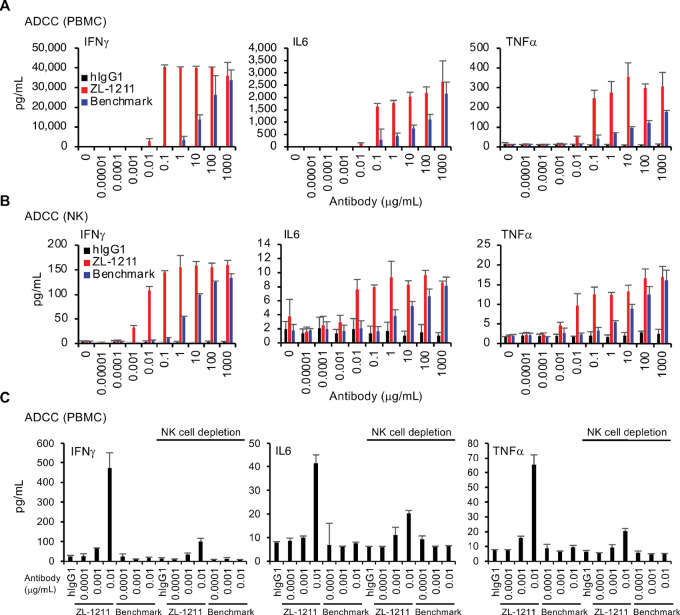
ZL-1211 induces a robust NK cell–mediated inflammatory response. **A,** ADCC was performed with SNU601 cells and human PBMC at E:T = 20:1 in presence of the indicated antibodies. IFNγ, IL6, or TNFα in the supernatants was measured by MSD. **B,** ADCC with purified NK cells at E:T = 1:1 was performed using SNU601 cells and then IFNγ, IL6, or TNFα in the supernatants was measured as described in **A**. **C,** NK cells were depleted from PBMC as described in Fig. 4B and the NK-depleted PBMC was used for ADCC with SNU601. IFNγ, IL6, or TNFα in the supernatants was measured as described in **A**.

NK cell triggers inflammatory responses including production of IFNγ and inflammatory cytokines upon activation, which further activates not only innate but also adaptive immunity including cytotoxic T cell (19–21). As NK cell was activated by ZL-1211 (Fig. 4C), we further evaluated whether activated NK cell can secrete IFNγ and inflammatory cytokines in response to ZL-1211. After performing ADCC for SNU601 with ZL-1211 or benchmark, supernatants were collected and cytokine levels in the supernatants were measured (Fig. 5A and B). Robust IFNγ, IL6, or TNFα production was induced during ADCC reaction with ZL-1211 when PBMC or purified NK cell was used as effector cell. Consistent with ADCC activity (Fig. 3B and 4A), ZL-1211 was more potent to induce the inflammatory cytokines than benchmark (Fig. 5A and B). When NK cell was depleted from PBMC, IFNγ, IL6, or TNFα production dramatically reduced (Fig. 5C; Supplementary Fig. S4A), indicating that NK cell is required for the cytokine production with ZL-1211. These results indicate that ZL-1211 induces robust inflammation with NK cell.

### ZL-1211 Exhibits Antigastric Tumor Efficacy in a Series of CLDN18.2-expressing PDX Models and Promotes NK-cell Infiltration into Tumor Microenvironment

**FIGURE 6:**
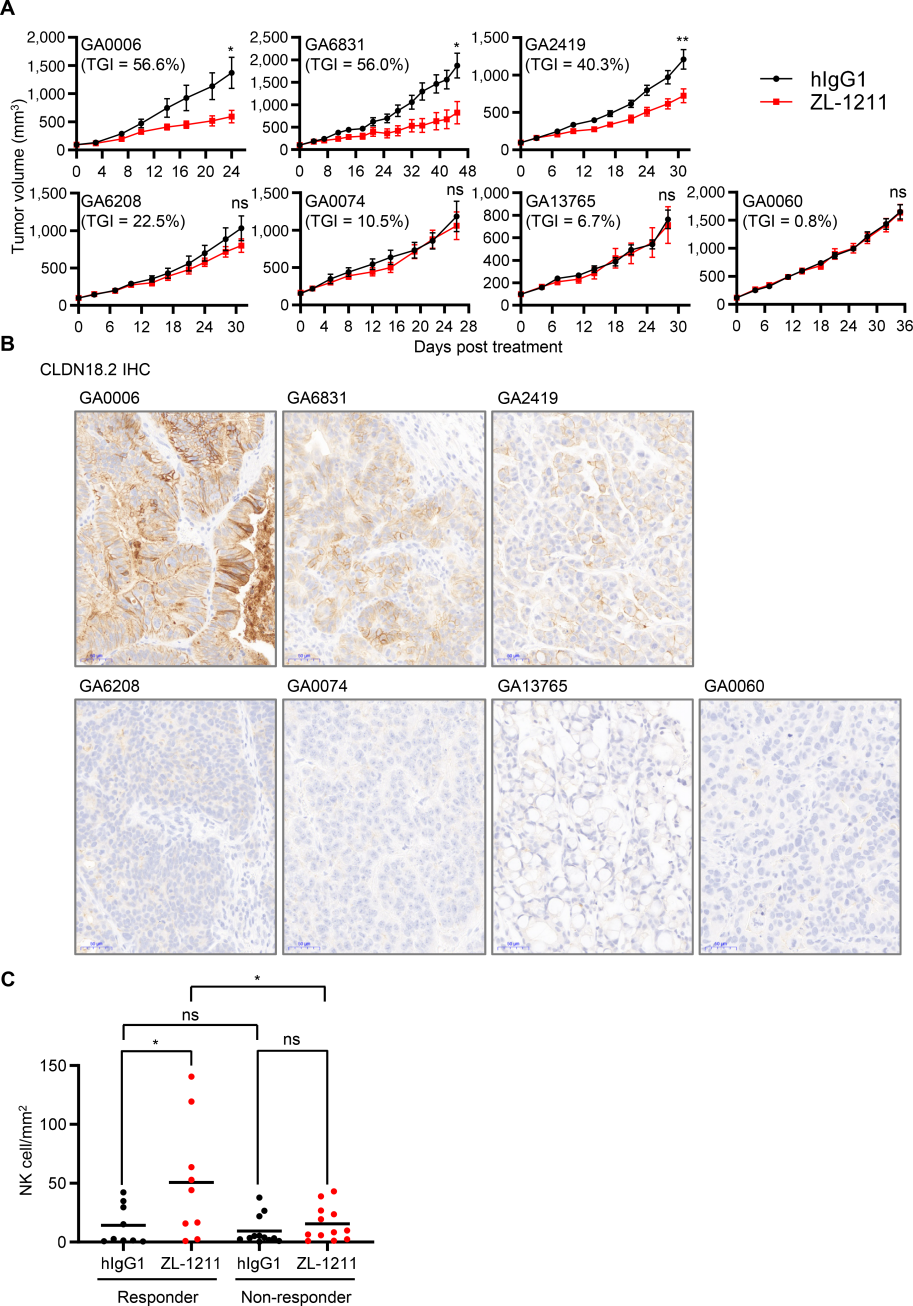
ZL-1211 exhibits antigastric tumor efficacy in a series of CLDN18.2-expressing PDX models and recruits NK cells into gastric tumor microenvironment. **A,** Gastric PDXs were subcutaneously inoculated into Balb/c nude mice (*n* = 10). The PDX-bearing mice were treated with 50 mg/kg of control antibody (hIgG1) or ZL-1211 by intraperitoneal route three times weekly. At the end of study, the tumor size was measured to calculate TGI. **B,** CLDN18.2 expression in tested PDXs was confirmed by CLDN18.2 IHC. DAB in brown. Hematoxylin: Nuclear counterstaining in blue. Scale bar, 50 μm. **C,** The tumor sections were stained with mouse NK marker, NKp46, and then image analysis was performed for presence of NK cell in tumor area. Three PDXs for each arm were tested for the image analysis. Data for three responders (GA0006, 6831, 2419) or nonresponders (GA6208, 0074, 13765, 0060) were combined to compare responder with nonresponder. * or ns indicates *P* < 0.05 or not significant, respectively.

Next, we tested ZL-1211 *in vivo* efficacy for gastric PDX because PDX model is known to have physiologically relevant tumor microenvironments and exhibit similar responses to anticancer agents as seen in the actual patient compared with cell line–derived tumor xenograft (CDX). Seven gastric PDXs were selected for ZL-1211 treatment based on RNA-seq data for *CLDN18.2* (Supplementary Fig. S5A). The PDXs were inoculated into Balb/c nude mice and treated with ZL-1211 three times a week (Fig. 6A). Among all the models tested, three PDXs (GA0006, 6831, 2419) responded to ZL-1211 treatment. The CLDN18.2 IHC analysis showed that all three responders had various levels of CLDN18.2 expression and non-responders (GA6208, 0074, 13765, 0060) were suspected to be CLDN18.2-negative (Fig. 6B; Supplementary Fig. S5B). These data clearly indicate that ZL-1211 can target only CLDN18.2-expressing tumors. In addition, the CLDN18.2 expression levels correlated with tumor growth inhibition (TGI) in view that GA2419 (TGI = 40.3%) with lower CLDN18.2-expression level was less sensitive to ZL-1211 treatment than GA6831 (TGI = 56.0%) and GA0006 (TGI = 56.6%) and, which showed medium- and high-expression levels of CLDN18.2, respectively (Fig. 6A and B). Taking advantage of the PDX model setting which allows us to detect the presence of ZL-1211 therapeutic antibody using anti-human IgG IHC, we also assessed the presence of ZL-1211 in various models (Supplementary Fig. S5B and S5C). Three responders showed markedly greater human IgG staining in ZL-1211 treatment arm than those in control arm (hIgG1). In contrast, non-responders showed comparable human IgG staining in ZL-1211 arm to their corresponding control arms. These results provided additional supportive data showing that ZL-1211 specifically binds CLDN18.2-expressing tumors, but not CLDN18.2-negative tumors, allowing CLDN18.2-targeted efficacy.

Next, we evaluated NK cell activation in the PDXs because ZL-1211 activates NK cells and induces NK cell–mediated inflammatory response (Fig. 4C and 5B). However, NK cell level is very low in tumors on Balb/c nude mouse model (Fig. 4D), we decided to perform IHC for NK cell infiltration instead of measuring inflammatory responses. To assess NK cell infiltration into tumor microenvironment and evaluate whether ZL-1211 changes tumor microenvironment immunologically, we performed anti-NKp46 IHC (Supplementary Fig. S5B and S5D) and NK cell density was determined by quantitative image analysis (Supplementary Fig. S5E). There is no statistically significant difference between control and ZL-1211 arms (*n* = 3) in NK-cell density for each PDX model (Supplementary Fig. S5E). However, when the 7 PDXs were divided to two groups by their response to ZL-1211 (responders: GA0006, 6831, 2419; nonresponders: GA6208, 0074, 13765, 0060), enhancement of NK-cell density after ZL-1211 treatment was observed only in responder group (*P* < 0.05; Fig. 6C). These results indicate that ZL-1211 recruits NK cells into tumor microenvironment, contributing to its antitumor efficacy.

## Discussion

In this study, we present ZL-1211, a novel, humanized monoclonal IgG1 antibody that targets CLDN18.2-expressing gastric cancers. Our data indicate that ZL-1211 inhibits tumor growth *in vitro* and *in vivo* by inducing tumor cell death through ADCC and CDC more potently than clinical leading benchmark. Furthermore, ZL-1211 induces robust NK-mediated inflammation through the production of IFNγ, IL6, or TNFα. Our findings also suggest that ZL-1211 treatment activates NK cells and increases perforin to destroy cancer cells. ZL-1211 treatment *in vivo* triggered NK cell recruitment into tumor microenvironment to inhibit gastric tumor growth. These findings clearly exhibit that ZL-1211 is a highly potent antibody for CLDN18.2-expressing gastric cancers and potentially other CLDN18.2-positive indications, including esophageal and pancreatic cancers.

Because CLDN18.2 expression is limited to malignant tissues such as stomach and pancreatic carcinomas, CLDN18.2 is an attractive tumor-specific marker for molecular targeted therapy. Not only mAb but also bispecific antibodies and CAR-T cell therapies targeting CLDN18.2 are being evaluated in clinical trials (2). An anti-CLDN18.2 mAb Zolbetuximab (IMAB362) is currently under evaluation in phase II and III clinical trials (4, 5). Early trial results have shown promising efficacy for gastric and esophageal tumor indications, however, significant clinical benefit was observed only in patients with high/medium CLDN18.2 expression (IHC3+/2+) in ≥70% of tumor cells but patients with CLDN18.2-low tumors (IHC1+) were excluded because of lack of clinical response (4). Compared with benchmark analog, ZL-1211 showed much greater potency for not only CLDN18.2-high/medium but also -low tumor models *in vitro* and *in vivo* (Figs. 2 and 3). Thus, ZL-1211 has the potential to drive more potent clinical activity in a wider spectrum of high- and low-CLDN18.2 expressing tumors than the leading clinical benchmark.

In FAST study (NCT01630083), approximately 50% of patients with gastric/gastroesophageal adenocarcinoma were eligible for IMAB362 treatment based on CLDN18.2 IHC test (define as ≥40% of tumor cells with 2+ or 3+ staining intensity; ref. 5). A group recently reported CLDN18.2 prevalence in gastric adenocarcinoma from Japanese patients (22). The group used same CLDN18.2 IHC antibody (clone 43-14A) used in the FAST study and applied FAST eligibility criterion to access CLDN18.2 positivity in the tumors. The authors reported 228 of 262 (87%) gastric tumors were CLDN18.2 positive but 135 of 262 (52%) could be eligible for IMAB362 treatment when the FAST criterion was applied. Thus, approximately 30% of patients with CLDN18.2-positive tumor could be excluded from the treatment. As we showed in this study, ZL-1211 exhibited better potency especially for CLDN18.2-low tumors than benchmark analog, suggesting that potentially up to 80% of gastric tumors may be eligible for ZL-1211 treatment. We will further evaluate treatment eligibility with ZL-1211 in our clinical trial and report in a subsequent article.

Although CLDN18.2 IHC test has been conducted for patient selection in clinical trials, CLDN18.2-positive criteria and cutoff for treatment eligibility are still under debate. The clone 43-14A is known to detect both CLDN18.2 and CLDN18.1 isoforms because the antibody was generated with the C-terminus of CLDN18.2 that is shared with CLDN18.1 (Supplementary Fig. S1A). Instead, several studies reported CLDN18.2-specific antibody (EPR19202) for IHC test that was generated with the N-terminus of CLDN18.2 (23–25). In the reports, authors showed that CLDN18.2 positivity by EPR19202 becomes lower compared with 43-14A, which may impact on the treatment eligibility and strategy. These reports suggest that standardization of CLDN18.2 positivity must be further evaluated and discussed in future studies. In our clinical trial, we have used CLDN18.2-specific antibody for the IHC test to specifically select CLDN18.2-positive tumor patients. The CLDN18.2 positivity and the cutoff from the clinical trial will be shared in a subsequent report.

NK cell is an innate immune effector cell that can lyse cancer cells without prior activation (19–21). Upon activation NK cell releases lysis granules including perforin and granzyme that cooperatively induce cancer cell apoptosis. In addition, NK cell recognizes antibody-coated cancer cells through CD16A (FcγRIIIa) expressed on NK cell to induce ADCC. Besides the direct cytotoxicity against cancer cells, NK cell can release inflammatory cytokines and IFNγ that further trigger activation of innate and adaptive immunity including cytotoxic CD8^+^ T cell in tumor microenvironment. As we showed in Fig. 1B, NK cell activation status correlates with prognosis in CLDN18.2-expressing gastric carcinomas, and, more specifically, a low level of NK cell activation portends a poor outcome in CLDN18.2-positive gastric cancers. Indeed, gastric tumors can evade from NK cell by inducing immunologically cold tumor microenvironment (26–28). Thus, we designed ZL-1211 to induce NK cell activation, which potentially improves treatment outcomes in patients with gastric tumor. The Fc-domain of ZL-1211 was mutated to enhance interaction with CD16A and, thus, increase ADCC activity (Fig. 1D). Strikingly, NK cell activation as well as robust cytokine production from the activated NK cells were observed upon ZL-1211 treatment (Figs. 4 and 5). Furthermore, *in vivo* ZL-1211 treatment triggers NK cell infiltration into tumor microenvironment not only in CLDN18.2-high but also -low gastric PDXs (Fig. 6). These results clearly suggest that ZL-1211 not only stimulates a robust ADCC response to lyse cancer cells but also acts as an “immune modulator” to enhance treatment outcomes by mounting inflammation in tumor microenvironment. Thus, combination treatment with immune checkpoint inhibitors may be warranted to further enhance ZL-1211 efficacy in the clinic.

Although ZL-1211 induces robust ADCC to cause tumor cell death with NK cell as we showed in this study, we think this preclinical study has two limitations. The first limitation is about benchmark analog. We generated benchmark analog based on disclosed amino acid sequence as described in Materials and Methods, but it may not be exactly same antibody for clinical grade IMAB362 due to lack of information regarding actual sequence, drug formulation, and modification. The second limitation is about mouse models. In this preclinical study, immunocompromised mouse models were used to evaluate ZL-1211 efficacy for human CDX or PDX models. While NK cell plays important roles in *in vivo* ZL-1211 efficacy, Balb/c nude mouse does not have functional T cell due to lack of thymus, meaning involvement of T cells in ZL-1211–induced tumor cell death cannot be evaluated with the mouse model. Because ZL-1211 induces robust inflammation with NK cell, T cell could be also activated by the produced cytokines in tumor microenvironment. To address whether T cell is involved in ZL-1211-medicated efficacy, we have tested syngeneic mouse models with ZL-1211 and will report in a subsequent article.

In conclusion, we developed potent anti-CLDN18.2 mAb ZL-1211 and demonstrated promising antitumor efficacy for not only CLDN18.2-high/medium but also -low expressing gastric tumors. ZL-1211 can induce robust ADCC and trigger inflammatory response in an NK cell–dependent manner to inhibit gastric tumor growth. Our preclinical data strongly indicate that ZL-1211 may be able to target CLDN18.2-low gastric tumors and has the potential to offer greater clinical benefit to a wider spectrum of CLDN18.2-positive gastric cancers.

## Supplementary Material

Supplementary Figure S1 Supplementary Figure 1 shows characterization of ZL-1211.

Supplementary Figure S2 Supplementary Figure 2 shows CLDN18.2 expression in MIA-PaCa2 clones and ZL-1211-induced CDC for the MIA-PaCa2 clones.

Supplementary Figure S3 Supplementary Figure 3 shows CLDN18.2 expression in gastric tumor cell lines and ZL-1211-induced CDC for the gastric tumor cell lines.

Supplementary Figure S4 Supplementary Figure 4 shows confirmation of NK depletion by flow cytometer and NK levels in spleen of the mouse models.

Supplementary Figure S5 Supplementary Figure 5 shows IHC for gastric PDX models with anti-CLDN18.2, human IgG, or NKp46 antibody.
