# Supplementary material for: ZL-1211 Exhibits Robust Antitumor Activity by Enhancing ADCC and Activating NK Cell–mediated Inflammation in CLDN18.2-High and -Low Expressing Gastric Cancer Models
Source: Cancer Res Commun. 2022 Sep 7;2(9):937–50. doi: 10.1158/2767-9764.CRC-22-0216 (PMC10010325; doi:10.1158/2767-9764.CRC-22-0216)
Supplement: Supplementary Figure S1 — Supplementary Figure 1 shows characterization of ZL-1211. [file crc-22-0216-s01.pdf]

Supplementary Figure 1

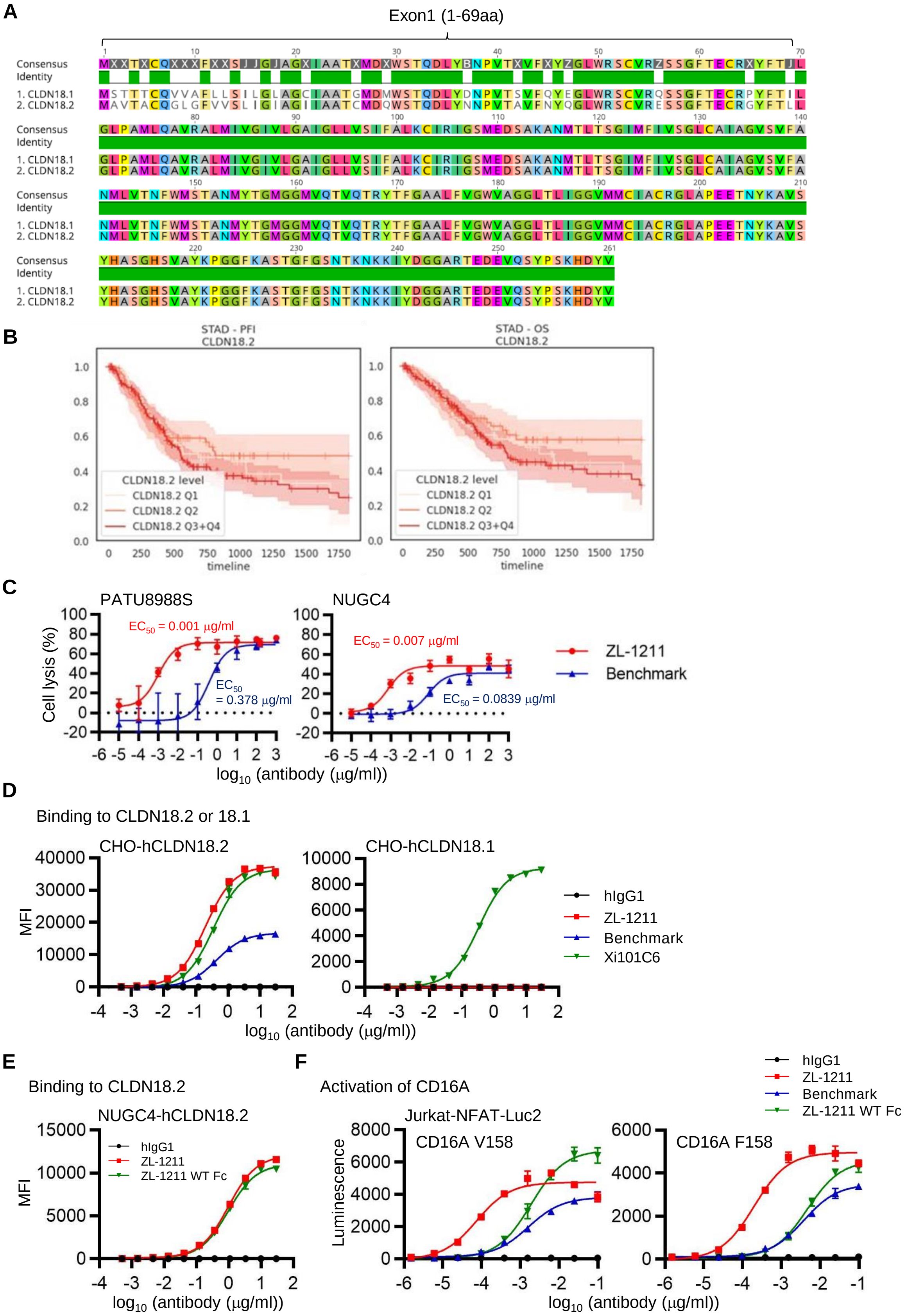

Supplementary Figure 1 (continued)

G

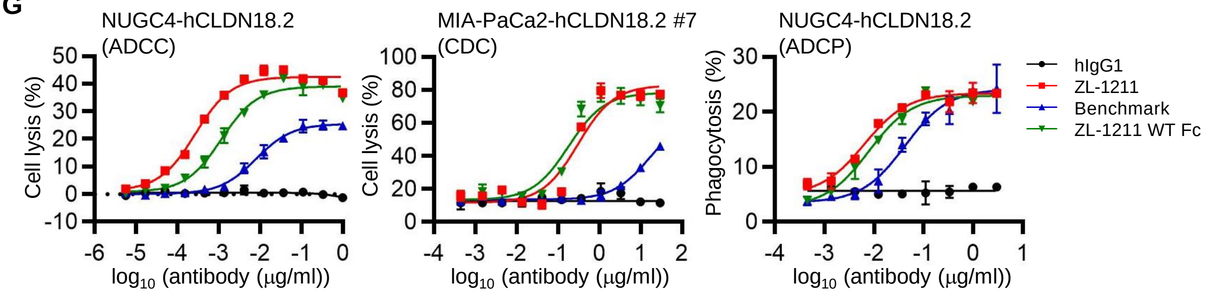

H

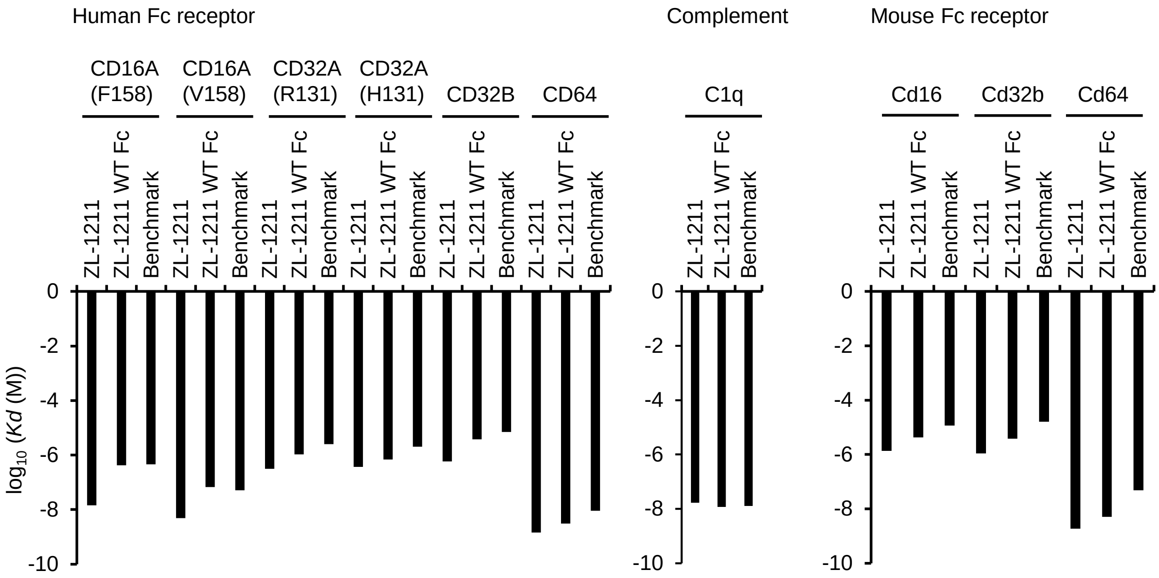

Supplementary Figure 1. Characterization of ZL-1211.

A, Comparison of human CLDN18 isoforms. **B,** Kaplan-Meier analysis of clinically-relevant CLDN18.2 subgroups in TCGA gastric cancers. **C,** Capability of benchmark analogue to induce ADCC for PATU8988S or NUGC4 with PBMC. **D,** Overexpressed human CLDN18.2 or CLDN18.1 in CHO cells was incubated with the indicated antibodies and the binding was confirmed by flow cytometry. **E,** NUGC4 cells overexpressed with CLDN18.2 were incubated with the indicated antibodies and the binding was confirmed by flow cytometry. ZL-1211 WT Fc doesn't have the Fc mutations. **F,** Jurkat T cells with a luciferase gene driven by nuclear factor of activated T cells (NFAT) promoter were used to measure affinity as well as activation upon ZL-1211 binding to CD16A. ZL-1211 induced more NFAT activation with both CD16A 158V/V (high affinity) and 158F/F (low affinity) than benchmark. **G,** CLDN18.2-overexpressed NUGC4 or MIA-PaCa2 cells were incubated with the indicated antibodies to measure ADCC with human PBMC (left), CDC with human serum (middle), or ADCP with induced macrophage (right). **H,** Affinity of ZL-1211, ZL-1211 WT Fc, or benchmark antibody to Fc receptors or complement.
