# Supplementary material for: ZL-1211 Exhibits Robust Antitumor Activity by Enhancing ADCC and Activating NK Cell–mediated Inflammation in CLDN18.2-High and -Low Expressing Gastric Cancer Models
Source: Cancer Res Commun. 2022 Sep 7;2(9):937–50. doi: 10.1158/2767-9764.CRC-22-0216 (PMC10010325; doi:10.1158/2767-9764.CRC-22-0216)
Supplement: Supplementary Figure S2 — Supplementary Figure 2 shows CLDN18.2 expression in MIA-PaCa2 clones and ZL-1211-induced CDC for the MIA-PaCa2 clones. [file crc-22-0216-s02.pdf]

Supplementary Figure 2

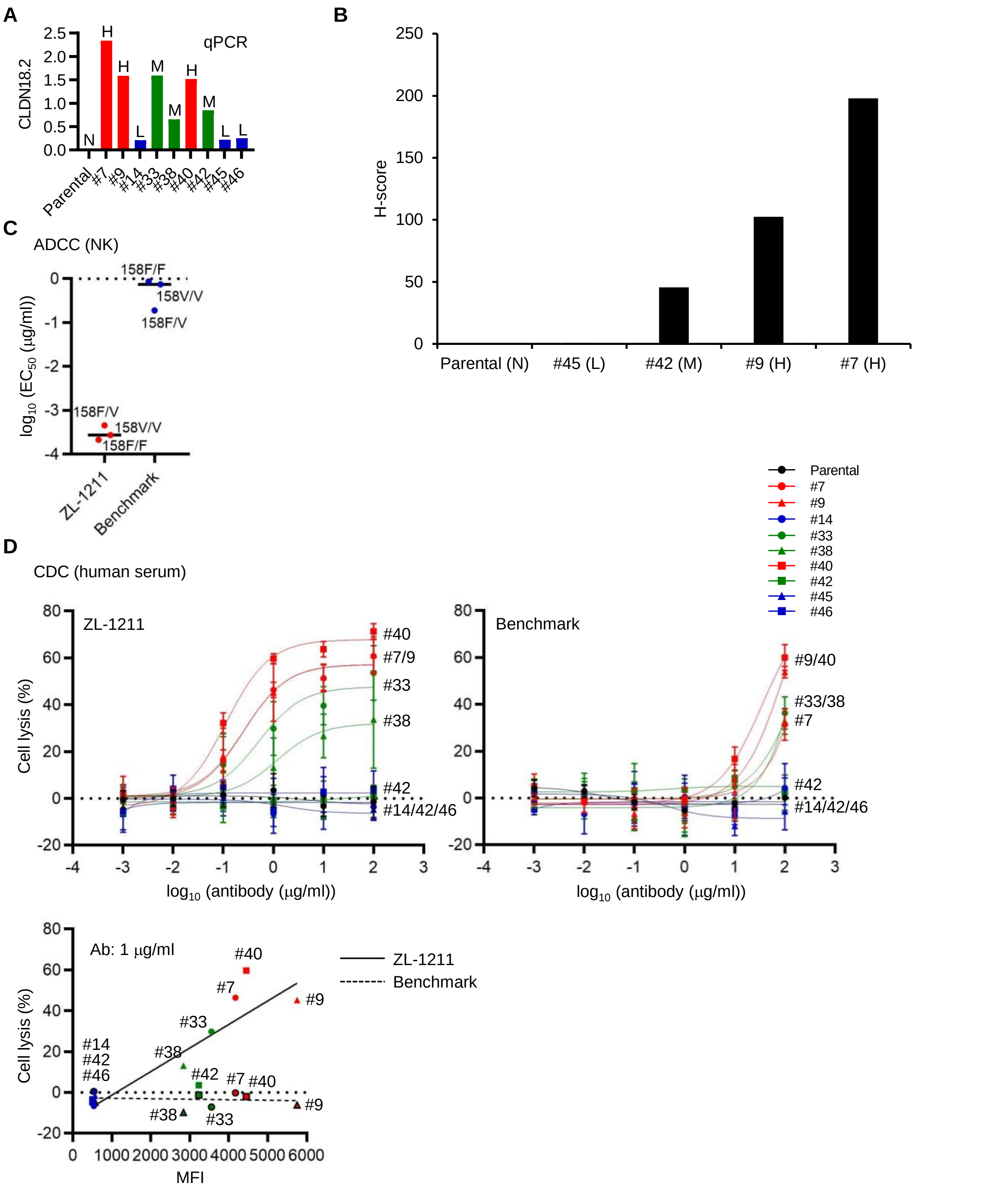

Supplementary Figure 2. CLDN18.2 expression in MIA-PaCa2 clones and CDC.

A, *CLDN18.2* expression in the isolated MIA-PaCa2 clones was determined by qPCR. GAPDH was used for normalization. **B,** CLDN18.2 expression was further confirmed by CLDN18.2 IHC. H-score was calculated for parental, #45, 42, 9, 7 clones. **C,** ADCC was performed with purified human NK cells against SNU601. **D,** The indicated clones were incubated with human AB serum in presence of ZL-1211 or benchmark antibody to measure CDC. The cell lysis levels for each clone were compared at 1 μg/ml of the antibody concentration (lower graph).
