# Supplementary material for: ZL-1211 Exhibits Robust Antitumor Activity by Enhancing ADCC and Activating NK Cell–mediated Inflammation in CLDN18.2-High and -Low Expressing Gastric Cancer Models
Source: Cancer Res Commun. 2022 Sep 7;2(9):937–50. doi: 10.1158/2767-9764.CRC-22-0216 (PMC10010325; doi:10.1158/2767-9764.CRC-22-0216)
Supplement: Supplementary Figure S3 — Supplementary Figure 3 shows CLDN18.2 expression in gastric tumor cell lines and ZL-1211-induced CDC for the gastric tumor cell lines. [file crc-22-0216-s03.pdf]

# Supplementary Figure 3

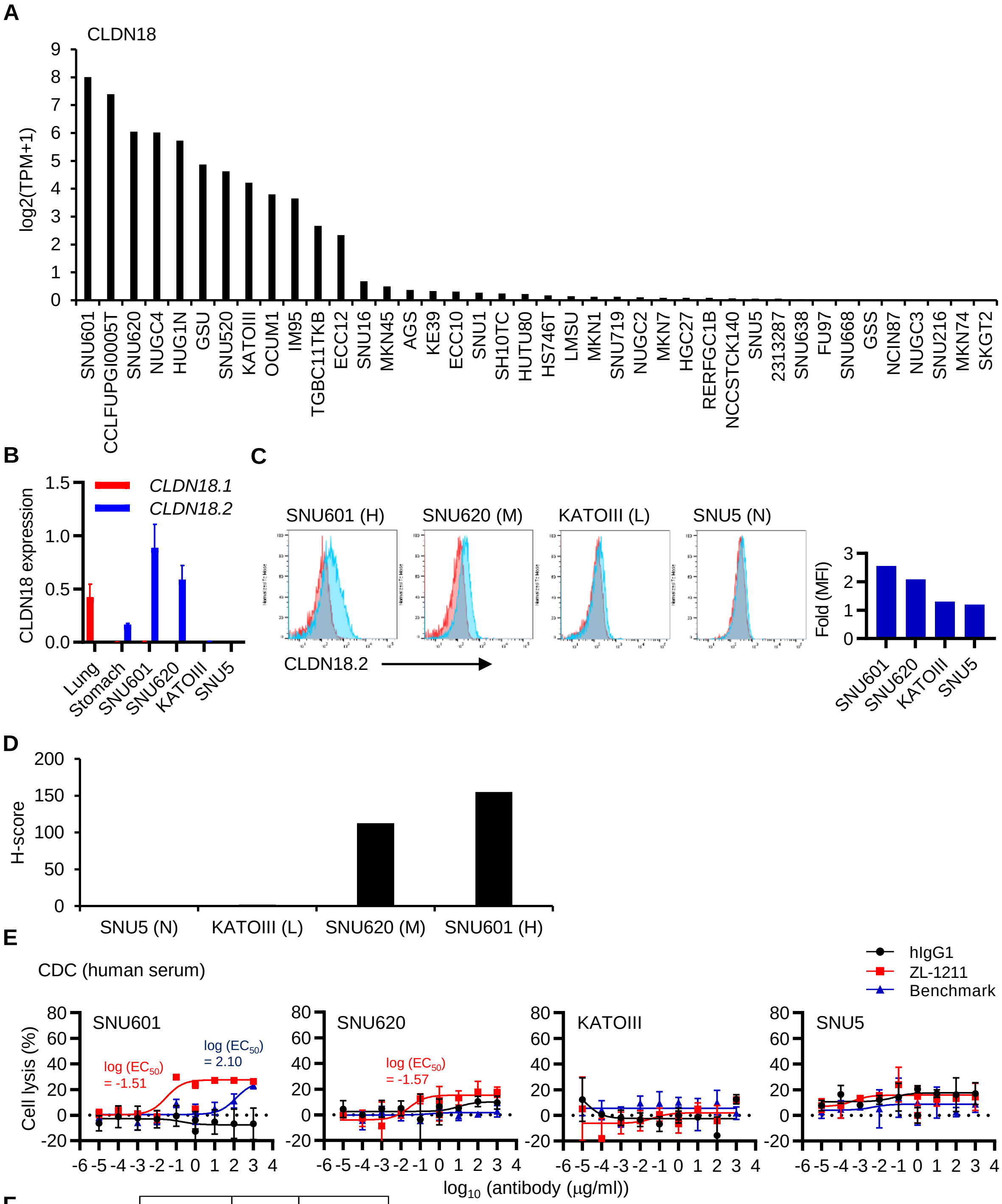


F

| | Dose | $C_{max}$ (µg/ml) | $AUC_{168h}$ (hr*µg/ml) |
|---|---|---|---|
| hIgG1 | 10 mg/kg | 99.1 | 11318 |
| ZL-1211 | 0.1 mg/kg | 1.31 | 109 |
| | 1 mg/kg | 16.7 | 1204 |
| | 10 mg/kg | 124 | 11921 |
| Benchmark | 0.1 mg/kg | 1.76 | 190 |
| | 1 mg/kg | 21.0 | 2351 |
| | 10 mg/kg | 161 | 20196 |

**Supplementary Figure 3. CLDN18.2 expression in gastric tumor cell lines and CDC.**

**A,** *CLDN18* expression in gastric tumor cell lines. The RNAseq data was downloaded from DepMap portal (https://depmap.org/portal/). **B,** *CLDN18.2* expression in the indicated gastric tumor cell lines was further confirmed by qPCR with CLDN18.1- or 18.2-specific primer. cDNA from human lung or stomach was used as positive control for CLDN18.1 or CLDN18.2, respectively. **C,** CLDN18.2 expression on cell surface was evaluated by flow cytometry. Histogram indicates CLDN18.2 expression by isotype control (pink) or ZL-1211 (blue). Fold indicates ratio of MFI for CLDN18.2 to MFI for isotype control. **D,** CLDN18.2 IHC was performed to confirm CLDN18.2 expression in the gastric tumor cell lines and H-score was calculated. **E,** The indicated gastric tumor cell lines were incubated with human AB serum in presence of ZL-1211 or benchmark to measure CDC. **F,** The serum concentration-time profiles of ZL-1211 or benchmark in SNU620 tumor-bearing mice were used to estimate the PK parameters.
