# Supplementary material for: ZL-1211 Exhibits Robust Antitumor Activity by Enhancing ADCC and Activating NK Cell–mediated Inflammation in CLDN18.2-High and -Low Expressing Gastric Cancer Models
Source: Cancer Res Commun. 2022 Sep 7;2(9):937–50. doi: 10.1158/2767-9764.CRC-22-0216 (PMC10010325; doi:10.1158/2767-9764.CRC-22-0216)
Supplement: Supplementary Figure S4 — Supplementary Figure 4 shows confirmation of NK depletion by flow cytometer and NK levels in spleen of the mouse models. [file crc-22-0216-s04.pdf]

Supplementary Figure 4

A

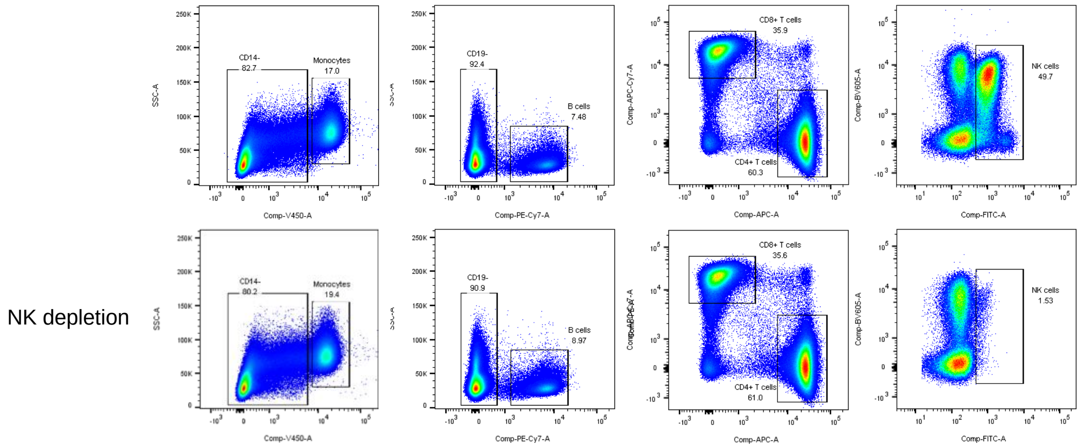

B

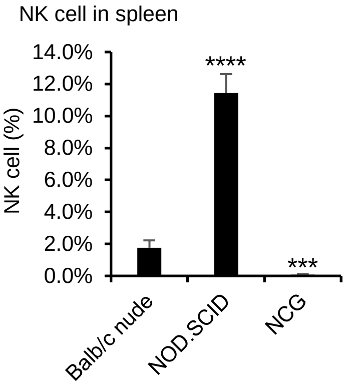

Supplementary Figure 4. NK cell depletion.

A, NK cells in human PBMC were depleted by anti-CD56 magnetic beads. NK cell depletion was confirmed by flow cytometry. Other immune cell levels such as monocyte, B cell, and T cell were not affected by the NK cell depletion. **B,** NK cell levels in spleen from SNU601 tumor-bearing Balb/c nude, NOD.SCID, or NCG mice were determined by flow cytometry as in **Figure 4D**.
