# Supplementary material for: ZL-1211 Exhibits Robust Antitumor Activity by Enhancing ADCC and Activating NK Cell–mediated Inflammation in CLDN18.2-High and -Low Expressing Gastric Cancer Models
Source: Cancer Res Commun. 2022 Sep 7;2(9):937–50. doi: 10.1158/2767-9764.CRC-22-0216 (PMC10010325; doi:10.1158/2767-9764.CRC-22-0216)
Supplement: Supplementary Figure S5 — Supplementary Figure 5 shows IHC for gastric PDX models with anti-CLDN18.2, human IgG, or NKp46 antibody. [file crc-22-0216-s05.pdf]

Supplementary Figure 5

A

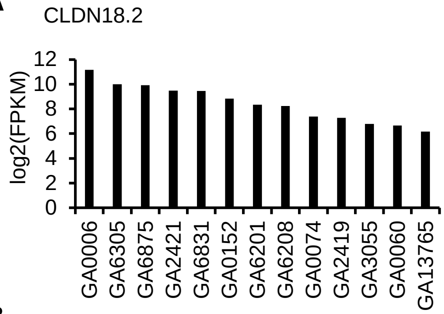

B

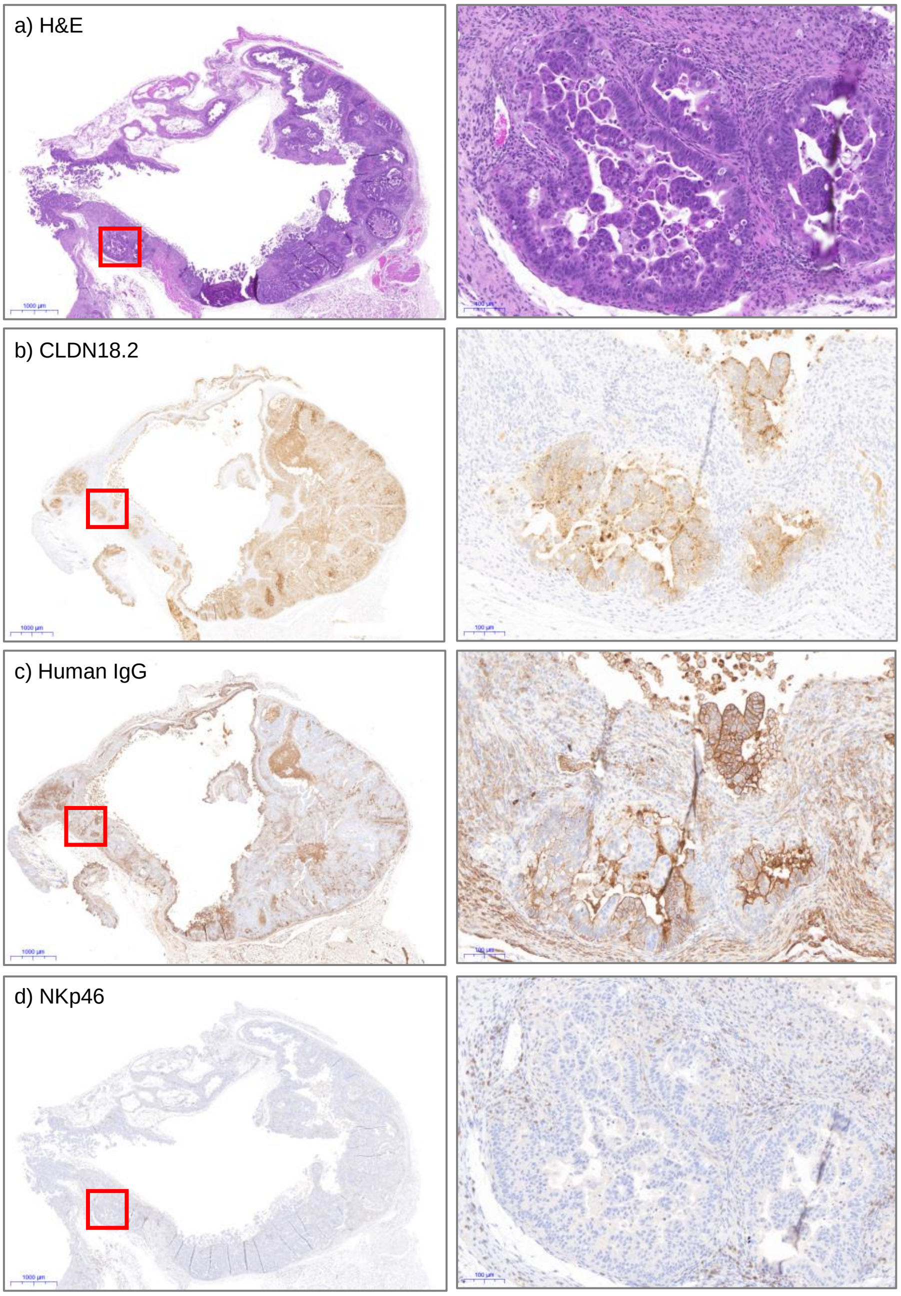

Supplementary Figure 5 (continued)

C

Human IgG IHC

	hIgG1			ZL-1211		
	1	2	3	1	2	3
GA0006	1-25%	1-25%	1-25%	26-50%	26-50%	26-50%
GA6831	1-25%	1-25%	1-25%	51-75%	51-75%	76-99%
GA2419	1-25%	<1%	<1%	76-99%	76-99%	76-99%
GA6208	<1%	<1%	<1%	1-25%	1-25%	1-25%
GA0074	<1%	<1%	<1%	1-25%	1-25%	26-50%
GA13765	<1%	<1%	<1%	<1%	<1%	<1%
GA0060	<1%	<1%	<1%	1-25%	1-25%	1-25%

D

NKp46 IHC

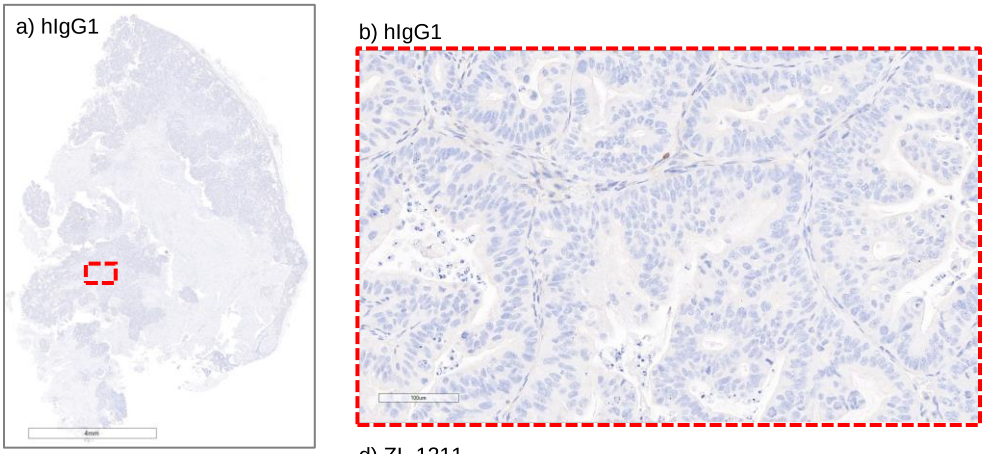

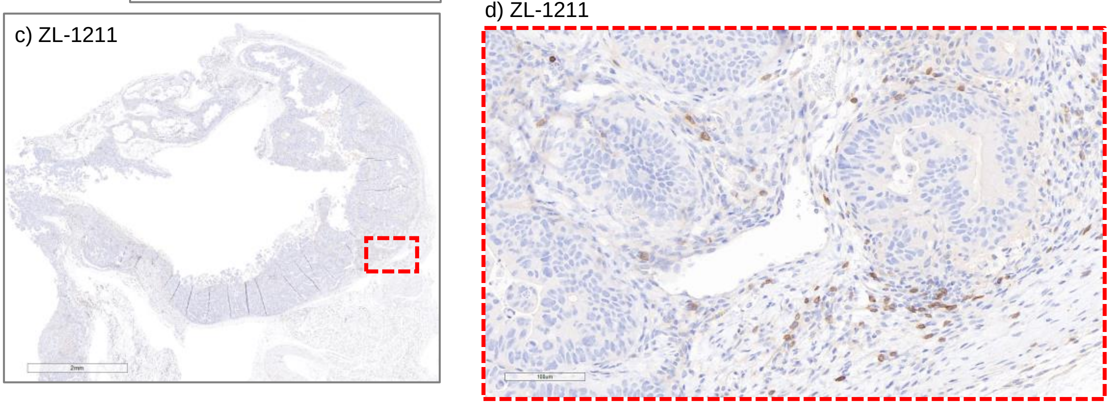

E

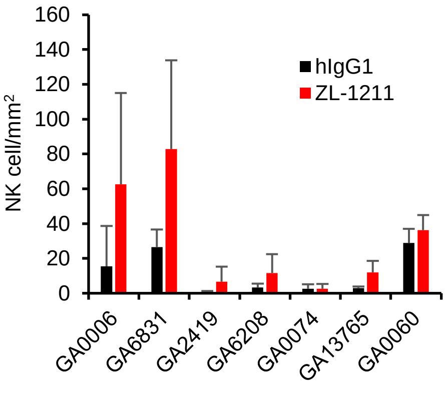

Supplementary Figure 5. IHC for gastric PDXs.

A, *CLDN18.2* expression in gastric PDX models. The RNAseq data was downloaded from Crown Bioscience (https://www.crownbio.com/). **B,** Representative IHC images of various markers using consecutive sections of the same tumor from ZL-1211 group of GA0006. Images on the right are images at higher magnifications for the areas indicated by red boxes. a) H&E. b) CLDN18.2 IHC shows membrane staining of tumor cells with non-specific staining in necrotic regions. c) Human IgG IHC shows membrane staining of some tumor cells. d) NKp46 IHC shows NKp46+ cells in tumor stroma. DAB in brown. Hematoxylin: Nuclear counterstaining in blue. **C,** Percentages of human IgG+ tumor cells per tumor section based on semi-quantitative analysis of anti-human IgG IHC images. Only membrane staining was counted as positive signal. **D,** Representative anti-NKp46 IHC images for tumors of GA0006. a) A representative tumor from IgG group. b) The boxed area in image a) at a higher magnification. c) A representative tumor from ZL-1211 group. d) The boxed area in image c) at a higher magnification. DAB in brown. Hematoxylin: Nuclear counterstaining in blue. **E,** NK cell density was determined by quantitative image analysis of anti-NKp46 IHC images.
